# Effect of Influenza Vaccination of Children on Infection Rate in Hutterite Communities: Follow-Up Study of a Randomized Trial

**DOI:** 10.1371/journal.pone.0167281

**Published:** 2016-12-15

**Authors:** Biao Wang, Margaret L. Russell, Lorraine Moss, Kevin Fonseca, David J. D. Earn, Fred Aoki, Gregory Horsman, Paul Van Caeseele, Khami Chokani, Mark Vooght, Lorne Babiuk, Richard Webby, Stephen D. Walter, Mark Loeb

**Affiliations:** 1 Department of Pathology and Molecular Medicine, McMaster University, Hamilton, Ontario, Canada; 2 Department of Community Health Sciences, University of Calgary, Calgary, Alberta, Canada; 3 Department of Microbiology and Infectious Diseases and Provincial Laboratory for Public Health, University of Calgary, Calgary, Alberta, Canada; 4 Department of Clinical Epidemiology and Biostatistics, McMaster University, Hamilton, Ontario, Canada; 5 Michael G. De- Groote Institute for Infectious Disease Research, McMaster University, Hamilton, Ontario, Canada; 6 Department of Mathematics and Statistics, McMaster University, Hamilton, Ontario, Canada; 7 Departments of Medicine, Medical Microbiology and Pharmacology, and Therapeutics, University of Manitoba, Winnipeg, Manitoba, Canada; 8 Saskatchewan Disease Control Laboratory, Regina, Saskatchewan, Canada; 9 Cadham Provincial Laboratory, Department of Medical Microbiology, University of Manitoba, Winnipeg, Manitoba, Canada; 10 Saskatchewan Health, Prince Albert Parkland Health Region, Prince Albert, Saskatchewan, Canada; 11 Saskatchewan Health, Five Hills Health Region, Moose Jaw, Saskatchewan, Canada; 12 Department of Agricultural, Food and Nutritional Science, University of Alberta, Edmonton, Canada; 13 St Jude Children’s Research Hospital and WHO Collaborating Center, Memphis, Tennessee, United States of America; 14 Department of Medicine, McMaster University, Hamilton, Ontario, Canada; McGill University Health Centre, CANADA

## Abstract

**Background:**

An earlier cluster randomized controlled trial (RCT) of Hutterite colonies had shown that if more than 80% of children and adolescents were immunized with influenza vaccine there was a statistically significant reduction in laboratory-confirmed influenza among all unimmunized community members. We assessed the impact of this intervention for two additional influenza seasonal periods.

**Methods:**

Follow-up data for two influenza seasonal periods of a cluster randomized trial involving 1053 Canadian children and adolescents aged 36 months to 15 years in Season 2 and 1014 in Season 3 who received the study vaccine, and 2805 community members in Season 2 and 2840 in Season 3 who did not receive the study vaccine. Follow-up for Season 2 began November 18, 2009 and ended April 25, 2010 while Season 3 extended from December 6, 2010 and ended May 27, 2011. Children were randomly assigned in a blinded manner according to community membership to receive either inactivated trivalent influenza vaccine or hepatitis A. The primary outcome was confirmed influenza A and B infection using RT-PCR assay. Due to the outbreak of 2009 H1N1 pandemic, data in Season 2 were excluded for analysis.

**Results:**

For an analysis of the combined Season 1 and Season 3 data, among non-recipients (i.e., participants who did not receive study vaccines), 66 of the 2794 (2.4%) participants in the influenza vaccine colonies and 121 of the 2301 (5.3%) participants in the hepatitis A colonies had influenza confirmed by RT-PCR, for a protective effectiveness of 60% (95% CI, 6% to 83%; P = 0.04); among all study participants (i.e., including both those who received study vaccine and those who did not), 125 of the 3806 (3.3%) in the influenza vaccine colonies and 239 of the 3243 (7.4%) in the hepatitis A colonies had influenza confirmed by RT-PCR, for a protective effectiveness of 63% (95% CI, 5% to 85%; P = 0.04).

**Conclusion:**

Immunizing children and adolescents with inactivated influenza vaccine can offer a protective effect among unimmunized community members for influenza A and B together when considered over multiple years of seasonal influenza.

**Trial Registration:**

Clinicaltrials.gov NCT00877396

## Introduction

Seasonal influenza can cause a large number of hospitalizations and deaths every year [1–3]. Pandemic outbreaks of influenza cause an increase in influenza illness, as was observed during the 2009 H1N1 pandemic outbreak where 190 countries were affected and where there were approximately 4500 deaths attributable to the outbreak [4]. Despite recommendations for annual influenza vaccination, influenza continues to pose a threat to public health. This is particularly relevant to groups, such as the elderly, whose ability to mount a robust response to the vaccine is reduced, rendering them at higher risk for complications [5].

Healthy children and adolescents appear to play an important role in the introduction and transmission of influenza into households and communities [6]. School-based trials and observational studies suggest that immunization of school-aged children may reduce the transmission of influenza [7–13]. There is—some evidence to suggest that vaccinating healthy children may lead to herd protection, where those who are not vaccinated may benefit [14, 15].

We conducted a cluster randomized trial in Canadian Hutterite communities in the 2008–2009 influenza season to assess the potential herd effect of giving healthy children and adolescents inactivated influenza vaccine [15]. We demonstrated a 60% herd protection from vaccinating children, that is, unimmunized participants were protected at a level similar to that provided to those who had been directly vaccinated [16]. This study was reported early because of the start of the 2009 pandemic. In this report, we describe the effect of vaccinating children during two additional influenza seasons, 2009–2010 and 2010–2011.

## Method

### Study design and participants

The design and results of the Hutterite community randomized trial for Season 1 (2008–2009) have been published previously [15]. In brief, we conducted a blinded cluster randomized controlled trial involving 49 Hutterite colonies in Alberta, Saskatchewan, and Manitoba. Study participants were categorized into two groups: healthy children and adolescents (i.e. who were eligible to be immunized for the study vaccines), and vaccine nonrecipients. Healthy children and adolescents were defined as those aged 36 months to 15 years, and without any underlying chronic conditions. These exclusion criteria included anaphylactic reaction to a previous dose of influenza vaccine; anaphylactic reaction to hepatitis A vaccine; and individuals with more than one condition that required medical follow-up or hospitalizations, such as: chronic heart or lung conditions, metabolic disorders, chronic kidney disease, compromised immune system, and conditions that compromise respiratory function. Children aged 6–23 months were not offered study vaccine as they were recommended by public health for routine publicly funded influenza vaccination. The rest of the participants were classified into vaccine nonrecipients.

Hutterite colonies were randomly assigned to influenza vaccinated group or hepatitis A vaccinated group by an independent statistician. The randomization was performed once at the beginning of the study for the colonies participating the following years, while new colonies were randomized when they were added to the study. Healthy children and adolescents in influenza vaccinated colonies were assigned to receive the recommended influenza vaccine. Healthy children and adolescents in hepatitis A vaccinated colonies were assigned to receive the hepatitis A vaccine in a manner that mimicked the influenza immunization schedule, in order to maintain blinding. Arrangements for shipment of vaccines were made by an intermediary clinical trials research organization that received the randomization code from the statistician.

Vaccinated children and nonrecipients of the study vaccine were assessed for signs and symptoms of influenza during the follow-up periods. The start date of the follow-up periods was defined by the start date of >1 laboratory-confirmed influenza case in 2 consecutive weeks from sentinel sites. The stop date of the follow-up periods was when no laboratory-confirmed influenza cases for 2 consecutive weeks on colonies in the health region. Surveillance was started at least 2 weeks after the last child in the health region's study colonies had been immunized. All study participants were assessed twice weekly by a research nurse using a standardized self-reported symptom or sign checklist, which was completed by a representative of a given family for all family members, and which was provided when the research nurse made a site visit. Nasopharyngeal specimens were obtained and information about the symptoms and their onset date were recorded when any of the participants reported new symptoms in the checklist. For more detailed information, refer to our previous report [15].

In season 1, the recommended inactivated trivalent seasonal influenza vaccines were A/Brisbane/59/2007(H1N1)–like virus, A/Brisbane/10/2007 (H3N2)–like virus, B/Florida/4/2006-like virus [17]. In Season 2 (2009–2010) the recommended inactivated seasonal vaccines were A/Brisbane/59/2007 (H1N1)-like virus, A/Brisbane/10/2007 (H3N2)-like virus and B/Brisbane/60/2008-like virus [18]. For Season 3 (2010–2011) the recommended inactivated seasonal vaccines were A/California/7/2009 (H1N1)-like virus, A/Perth/16/2009 (H3N2)-like virus and B/Brisbane/60/2008-like virus [19].

The recruitment of participants took place between September and December -. The follow-up period for Season 1 extended from December 28, 2008 to June 23, 2009, from November 19, 2009 to April 25, 2010 for Season 2, and from December 6, 2010 to May 27, 2010 for Season 3.

### Ethics Approval

Our research protocol was approved by McMaster University Research Ethics Review Board. We obtained written informed consent from each participant at the start of each study year. Individuals aged 16 years and older signed by themselves. For enrollees aged 15 years and younger, a signature was provided on the consent form by a parent, guardian or family representative.

### Outcomes

The primary outcome of the study was laboratory-confirmed influenza A and B in nonrecipients using a real-time reverse transcriptase polymerase chain reaction (RT-PCR) assay. Influenza was confirmed in participants—including those who did or did not receive the vaccine—with at least 2 symptoms (i.e., fever defined as temperature ≥38°C, cough, nasal congestion, sore throat, headache, sinus problems, muscle aches, fatigue, ear ache or infection, or chills) by detection of viral RNA in respiratory samples using the Centers for Disease Control and Prevention Human Influenza Virus Real-time RT-PCR Detection and Characterization Panel, which targets the matrix gene for influenza A and nonstructural gene for influenza B.

We also assessed antimicrobial prescriptions, influenza-like illness (defined as temperature ≥ 38.0° Celsius and cough), medically attended visits for respiratory illness, school or work related absenteeism, emergency department visits, hospital admissions, and deaths [15].

All vaccinated participants were observed for 15 minutes immediately after vaccination. Participants were also assessed for adverse events for five days following vaccination. Passive surveillance for adverse reactions to the vaccine was implemented throughout the study period.

### Statistical analysis

We used a Cox proportional hazards regression model with robust sandwich variance estimates [20] to assess vaccine effectiveness for each season. In the Cox proportional hazards regression model, the start time was the surveillance start time for each season; the end time was symptom onset time for participants that had outcomes and surveillance end time for these who did not have outcome. During the three study periods, each specific outcome for a study participant within a season was only counted once to avoid lack of independence associated with counting multiple outcomes. For example, suppose a participant had been detected to have two episodes of H1N1 infection in Season 1, we just counted the first occurrence in the analysis in that season. All analyses adopted an intention-to-treat analysis plan and all original randomization treatment assignments were kept. Vaccine effectiveness was estimated using the hazard ratio (HR) [(1-HR)*100].

We assessed the overall vaccine effectiveness for multiple seasons through a nested frailty Cox proportional hazards regression model, with the consideration that most participants were enrolled in the study for more than one season [21, 22].

For the secondary outcomes, we used generalized estimating equations to account for membership in the randomized clusters with the logit-link function for dichotomous variables [23]. To avoid lack of independence associated with counting multiple outcomes, each specific outcome within a season in a participant was only counted once in our analyses. Outcomes were analyzed on an intention-to-treat basis. All p values and 95% confidence intervals were calculated as two-sided. Differences with p< 0.05 were considered statistically significant. Statistical analyses were performed with R (version 3.2.2) software [24]. A data safety and monitoring board provided oversight.

## Results

### Participants

**Fig 1:**
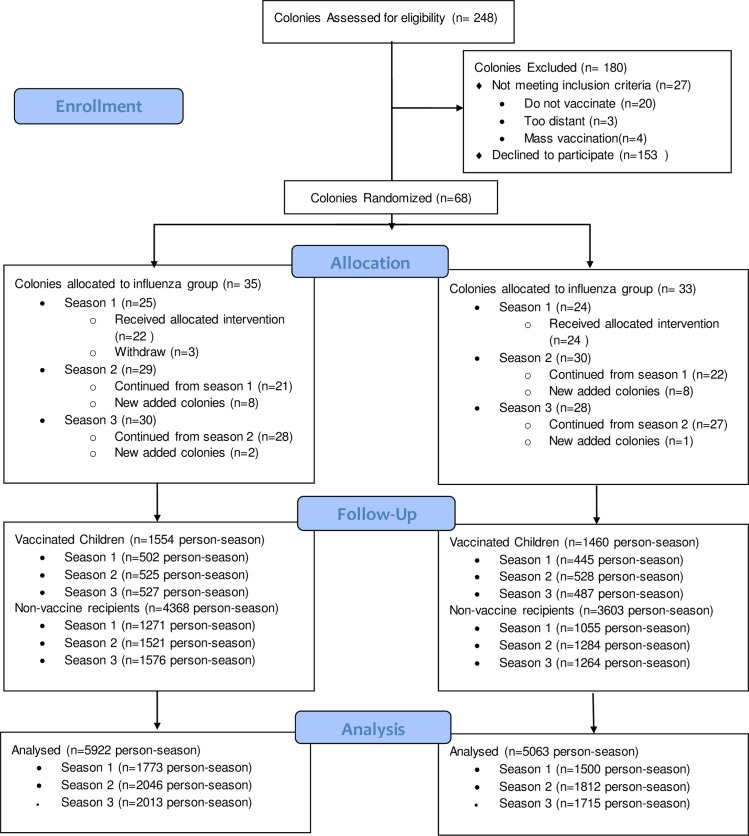
Participant flow.

A total of 4,640 participants from 65 colonies were enrolled over the three seasons (Fig 1). The results for Season 1 have been described previously [15]. In summary, 187 colonies in Alberta, Manitoba, and Saskatchewan were approached about the study. Of these, 46 participated in the study and 22 colonies were assigned to the influenza vaccinated group and 24 to the hepatitis A vaccinated group. In Season 2, one colony from the influenza vaccinated group (209 participants) and two from the hepatitis A vaccinated group (246 participants) chose not to re-enroll, and 8 new colonies (482 participants) were assigned to the influenza vaccinated group and 8 new colonies (558 participants) assigned to the hepatitis A group. In Season 3, again one influenza vaccinated group colony (157 participants) and three hepatitis A vaccinated group colonies (249 participants) did not re-enroll; two new colonies (214 participants) were assigned to the influenza vaccinated group, and one new colony (188 participants) was assigned to the hepatitis A vaccinated group. The intention-to- treat analysis therefore included data from 65 colonies (32 influenza vaccinated group and 33 hepatitis A vaccinated group) and 4, 640 unique participants. Characteristics of the enrolled colonies and participants were similar between the two groups (Table 1). Because participants were re-enrolled each influenza season, the final analysis was based on the total number of participants enrolled in each season. There were 5,922 participant-seasons for the influenza vaccinated group (1,773 in Season 1, 2,046 in Season 2, 2,103 in Season 3). There were 5,063 participant-seasons for the hepatitis A vaccinated group (1,500 in Season 1, 1,812 in Season 2, 1,751 in Season 3). Over the three-year study period, the average study vaccine coverage among healthy children (vaccinated children/total number of healthy children aged 3 to 15 years enrolled) was 76.5% for the influenza vaccinated group colonies and 81.3% for the hepatitis A vaccinated group colonies. For Season 1 and Season 3 combined, the average study vaccine coverage among healthy children was 76.9% for the influenza vaccinated group colonies and 81.4% for the hepatitis A vaccinated group colonies.

**Table 1:** Baseline Characteristics of All Study Participants in 65 Colonies (32 in Influenza Vaccinated Group and 33 in Hepatitis A Vaccinated group) at First Entry to Study.

	Influenza Vaccine Colonies	Hepatitis A Vaccine Colonies
	N = 2410	N = 2230
Mean age of study participants (± standard deviation)	25.6 ± 20.4	25.7 ± 20.1
Mean number of children aged less than 2 years per colony (± standard deviation)	7.9 ± 4.5	6.5 ± 4.5
Mean number of participants aged greater than or equal to 65 years per colony (± standard deviation)	4.6 ± 2.3	4.6 ± 3.0
Female sex–no. (%)	1361 (56.4)	1221 (54.8)
Vaccinated against influenza ^a^ –no. (%)	267 (11.1)	210 (9.4)
≥ 1 Co-existing condition–no. (%)	283 (11.7)	275(12.3)
Heart or lung disorders combined	151 (6.3)	126 (5.7)
Blood disorders combined	19 (0.8)	23 (1)
Swallowing or choking problems	12 (0.5)	12 (0.5)
Children and adolescents with conditions that require treatment for long periods with acetylsalicylic acid	0 (0)	2 (0.1)
Chronic metabolic diseases combined	101 (4.2)	105 (4.7)
Kidney or liver disease / dysfunction combined	9 (0.4)	16 (0.7)
Cancer, immunodeficiency, immunosuppression combined	23 (1.0)	24 (1.1)
Pregnancies	38 (1.6)	36 (1.6)
Clusters ^b^		
Mean number of enrolled participants per cluster (± standard deviation)	80.4 ± 28.2	71.9 ± 24.1
Study vaccine recipients	627	575
Mean number of study vaccine recipients per cluster (± standard deviation)	20.9± 8.5	18.5± 8.3

^a^ Refers to individuals who received influenza vaccine at baseline; the denominator excludes children who were immunized as part of the study.

^b^ Refers to participants at entry and during follow-up. Each colony is a cluster.

### Outcomes

#### Primary outcome–Vaccine protective effectiveness

Influenza A or B was detected in 172 (2.7%) persons in the influenza vaccinated group over the three years of the study compared to 263 (5.2%) persons in the hepatitis A vaccinated group (Table 2).

**Table 2:** Influenza A or B Detected in All Participants in the Study ^a^.

		Influenza Vaccine Colonies	Hepatitis A Vaccine Colonies
**RT-PCR confirmed influenza A or B**			
**All seasons**			
**Number of participants**		5922	5063
**All influenza–no.(%)**		172 (2.7%)	263 (5.2%)
**Influenza A–no.(%)**		85 (1.5%)	186 (3.7%)
	Seasonal H1N1– no.	2	41
	Seasonal H3N2– no.	33	117
	pH1N1– no.	45	24
	Unknown influenza A -no	5	4
**Influenza B–no.(%)**		87 (1.5%)	79 (1.6%)
**Season 1 (2008–2009)**			
**Number ofparticipants**		1773	1500
**All influenza–no.(%)**		95 (5.4%)	159 (10.6%)
**Influenza A–no.(%)**		46 (2.6%)	97 (6.5%)
	Seasonal H1N1– no.	2	41
	Seasonal H3N2– no.	25	56
	pH1N1– no.	15	--
	Unknown influenza A–no.	4	--
**Influenza B–no.(%)**		49 (2.8%)	62 (4.1%)
**Season 2 (2009–2010)**			
**Number of participants**		2046	1812
**All influenza–no.(%)**		30 (1.5%)	24 (1.3%)
**Influenza A–no.(%)**		30 (1.5%)	24 (1.3%)
	Seasonal H1N1– no.	--	--
	Seasonal H3N2– no.	--	--
	pH1N1– no.	30	24
	Unknown influenza A–no.	--	--
**Influenza B–no.(%)**		--	--
**Season 3 (2010–2011)**			
**Number of participants**		2103	1751
**All influenza–no.(%)**		47 (2.3%)	80 (4.6%)
**Influenza A–no.(%)**		9 (0.4%)	65 (3.7%)
	Seasonal H1N1– no.	--	--
	Seasonal H3N2– no.	8	61
	pH1N1– no.	--	--
	Unknown influenza A–no.	1	4
**Influenza B–no.(%)**		38 (1.8%)	17 (1%)

^a^ The sum of Influenza A and Influenza B is greater than All Influenza when participants were co-infected with both Influenza A and Influenza B. There were two persons in Season 3 detected with both influenza H3N2 and influenza B in the hepatitis A vaccinated group.

Of these, 85(1.5%) in the influenza vaccinated group and 186 (3.7%) in the hepatitis A vaccinated group were influenza A, while 87 (1.5%) were influenza B in the influenza vaccinated group compared to 79 (1.6%) in the hepatitis A vaccinated group. There were two persons in Season 3 detected with both influenza H3N2 and influenza B in the hepatitis A vaccinated group.

Of the 85 persons in whom influenza A was detected in the influenza vaccinated group, 2 (2.4%) were seasonal influenza H1N1 (from the first season); 33 (38.8%) were seasonal influenza H3N2 (25 from the first and 8 from the third season); 45 (52.9%) were pH1N1 (15 from the first and 30 from the second season); and 5(5.9%) were unknown influenza A (4 from the first and 1 from the third season). Of the 186 infected with influenza A detected in the hepatitis A vaccinated group, 41(22.0%) were seasonal influenza H1N1 (from the first season); 117 (62.9%) were seasonal influenza H3N2 (56 from the first season and 61 from the third season); 24 (12.9%) were pH1N1 (from the second season); and 4 (2.1%) were unknown influenza A (from the third season).

Of the 87 influenza B detected in the influenza vaccinated group, 49 (56.3%) were from the first season and 38 (43.7%) from the third season. Of the 79 influenza B detected in the hepatitis A vaccinated group, 62 (80.5%) were from the first season and 17 (19.5%) from the third season.

In season 1, there was a vaccine match for influenza A (A/Brisbane/59/2007[H1N1]–like virus and A/Brisbane/10/2007[H3N2]–like virus). There was a lineage mismatch for influenza B between influenza vaccine (B/Florida/4-like virus; Yamagata lineage) and the circulating strain (B/Brisbane/60/2008-like virus; Victoria lineage) [17].

In Season 2, a novel strain of the pandemic influenza virus A/H1N1 (pH1N1 2009) emerged. The pandemic had two waves in Canada extending from April to August of 2009 for the first wave and from September, 2009 to January, 2010 for the second wave [18]. Because of the pandemic, the influenza season extended from April 2009 until the winter of 2010. It was not possible to evaluate vaccine effectiveness when Hutterites were followed during 2009 to 2010. No surveillance was done over the summer and there was a delay in H1N1 vaccination (i.e., Health Canada authorized the sale of adjuvanted and unadjuvanted H1N1 2009 vaccine on October 21 and November 12 of 2009 respectively) [18].

In season 3, there was a vaccine match for influenza A (A/California/7/2009[H1N1]–like virus and A/Perth/16/2009[H3N2]–like virus). Although there was also a lineage match for influenza B between vaccine influenza strain and the predominant circulating strain (B/Brisbane/60/2008-like virus; Victoria lineage), there were a small number of influenza B isolates characterized as another lineage (B/Wisconsin/01/2010-like virus; Yamagata lineage) not covered by the vaccine [19].

In season 1, laboratory-confirmed influenza was detected in 125 nonrecipients: 45 (3.6%) in the colonies assigned to the influenza vaccine group (2 seasonal H1N1, 17 seasonal H3N2, 6 pH1N1, 4 unknown influenza A, and 16 influenza B by RT-PCR) and 80 (7.6%; 24 seasonal H1N1, 36 seasonal H3N2, and 24 influenza B by RT-PCR) in colonies assigned to hepatitis A. The level of indirect vaccine protective effectiveness among nonrecipients was 61% (95% CI, 7% to 83%; P = 0.03) (Table 3). Table 4 shows the level of vaccine effectiveness among all study participants. This analysis excludes the 15 cases of 2009 pH1N1 in season 1. The level of vaccine protective effectiveness among all study participants was 59% (95% CI, 4% to 82%; P = 0.04 (Table 4).

**Table 3:** Protective Effectiveness on Nonrecipients of Immunizing Children and Adolescents With Influenza Vaccine.

	Influenza Vaccine Colonies	Hepatitis A Vaccine Colonies	Hazard Ratio (95% CI)	Protective Effectiveness	P value
**RT-PCR -confirmed Influenza**					
**All Influenza** ^**a**^					
**All Seasons** ^**b**^	84/4300 (2%)	132/3581 (3.7%)	0.46 (0.21 to 1.02)	54 (-2 to 79)	0.0550
**Season 1&3**	66/2800 (2.4%)	121/2301 (5.3%)	0.4 (0.17 to 0.94)	60 (6 to 83)	**0.0360**
Season 1 (2008–2009) ^c^^,d^	39/1271 (3.1%)	80/1055 (7.6%)	0.39 (0.17 to 0.93)	61 (7 to 83)	0.0334
Season 2 (2009–2010) ^c^	18/1521 (1.2%)	11/1284 (0.9%)	1.38 (0.28 to 6.74)	-38 (-574 to 72)	0.6920
Season 3 (2010–2011) ^c^	29/1573 (1.8%)	41/1253 (3.3%)	0.56 (0.2 to 1.56)	44 (-56 to 80)	0.2660
**Influenza A**					
**All Seasons** ^**b**^	47/4300 (1.1%)	107/3581 (3%)	0.3 (0.11 to 0.8)	70 (20 to 89)	0.0170
**Season 1&3**	29/2800 (1%)	96/2301 (4.2%)	0.23 (0.07 to 0.75)	**77 (25 to 93)**	**0.0150**
Season 1 (2008–2009) ^c^^,^^d^	23/1271 (1.8%)	60/1055 (5.7%)	0.31 (0.09 to 1.04)	69 (-4 to 91)	0.0580
Season 2 (2009–2010) ^c^	18/1521 (1.2%)	11/1284 (0.9%)	1.38 (0.28 to 6.74)	-38 (-574 to 72)	0.6920
Season 3 (2010–2011) ^c^	7/1573 (0.4%)	36/1253 (2.9%)	0.15 (1.92 to 22.42)	**85 (48 to 96)**	**0.0027**
**Influenza B**					
**All Seasons** ^**b**^	37/4300 (0.9%)	27/3581 (0.8%)	1.05 (0.29 to 3.82)	-5 (-282 to 71)	0.9400
**Season 1&3**	37/2800 (1.3%)	27/2301 (1.2%)	1.05 (0.29 to 3.72)	-5 (-272 to 71)	0.9500
Season 1 (2008–2009) ^c^^,^^d^	16/1271 (1.3%)	20/1055 (1.9%)	0.66 (0.17 to 2.49)	34 (-149 to 83)	0.5380
Season 2 (2009–2010) ^c^	0/1521 (0)	0/1284 (0)	--	--	--
Season 3 (2010–2011) ^c^	22/1573 (1.4%)	5/1253 (0.4%)	2.5 (0.67 to 9.28)	-150 (-828 to 33)	0.1700

Abbreviations: CI confidence interval; RT-PCR, real-time polymerase chain reaction

^a^ The sum of Influenza A and Influenza B is greater than All Influenza when participants were co-infected with both Influenza A and Influenza B. All Influenza hazard ratios were calculated using the participants’ first infection with influenza.

^b^ The denominator is a sum of individuals enrolled each year. The fact that an individual could contribute to more than one year was taken into account in the analysis using a nested frailty model. In the nested frailty model, individuals are nested within colonies. To maintain the nested data structure, individuals that changed colony during the study periods were excluded from the pooled analysis. There were 32 participants changed their colony during the study period. Of these, 28 cases were originally from five influenza vaccinated colonies and four cases from three hepatitis A vaccinated colonies. One individual (31 years old) was infected by influenza A H3N2 in season 1 and one individual (38 years old) was infected by influenza B in season 3. They both were from the influenza vaccinated colonies.

^c^ A robust sandwich variance estimator was used with Cox proportional hazards to adjust for membership in the randomized colony.

^d^ 15 cases of 2009 H1N1 were detected from May 22 to June 4,2009 in the study population. All these 15 cases were in influenza vaccinated group and excluded from the analysis.

**Table 4:** Protective Effectiveness of Immunizing Children and Adolescents With Influenza Vaccine on All Participants.

		Influenza Vaccine Colonies	Hepatitis A Vaccine Colonies	Hazard Ratio (95% CI)		Protective Effectiveness	P value
**RT-PCR -confirmed Influenza**							
**All Influenza** ^**a**^							
**All Seasons** ^**b**^	155/5841 (2.7%)		263/5051 (5.2%)		0.41 (0.17 to 0.98)	59 (2 to 83)	0.0440
**Season 1&3**	125/3821 (3.3%)		239/3243 (7.4%)		0.37 (0.15 to 0.95)	63 (5 to 85)	0.0390
Season 1 (2008–2009) ^c^^,^^d^	80/1773 (4.6%)		159/1500 (10.6%)		0.41 (0.18 to 0.96)	59 (4 to 82)	0.0409
Season 2 (2009–2010) ^c^	30/2046 (1.5%)		24/1812 (1.3%)		1.11 (0.22 to 5.47)	-11 (-447 to 78)	0.9020
Season 3 (2010–2011) ^c^	47/2103 (2.2%)		80/1751 (4.6%)		0.48 (0.17 to 1.32)	52 (-32 to 83)	0.1550
**Influenza A**							
**All Seasons** ^**b**^	69/5841 (1.2%)		186/5051 (3.7%)		0.23 (0.09 to 0.62)	77 (38 to 91)	0.0037
**Season 1&3**	39/3821 (1.0%)		162/3243 (5.0%)		0.18 (0.05 to 0.57)	82 (43 to 95)	0.0037
Season 1 (2008–2009) ^c^^,^^d^	31/1773 (1.8%)		97/1500 (6.5%)		0.26 (1.04 to 13.75)	74 (4 to 93)	0.0435
Season 2 (2009–2010) ^c^	30/2046 (1.5%)		24/1812 (1.3%)		1.11 (0.22 to 5.47)	-11 (-447 to 78)	0.9020
Season 3 (2010–2011) ^c^	9/2103 (0.4%)		65/1751 (3.7%)		0.11 (0.03 to 0.37)	89 (63 to 97)	0.0003
**Influenza B**							
**All Seasons**^**b**^	86/5841 (1.5%)		79/5051 (1.5%)		1.31 (0.28 to 6.19)	-31 (-519 to 72)	0.7400
**Season 1&3**	86/3821 (2.3%)		79/3243 (2.4%)		1.28 (0.28 to 5.8)	-28 (-480 to 72)	0.7500
Season 1 (2008–2009) ^c^^,^^d^	49/1773 (2.8%)		62/1500 (4.1%)		0.67 (0.17 to 2.69)	33 (-169 to 83)	0.5720
Season 2 (2009–2010)^c^	0/2046 (0)		0/1812 (0)		--	--	--
Season 3 (2010–2011) ^c^	38/2103 (1.8%)		17/1751 (1%)		1.86 (0.41 to 8.48)	-86 (-748 to 59)	0.4220

Abbreviations: CI confidence interval; RT-PCR, real-time polymerase chain reaction

^a^ The sum of Influenza A and Influenza B is greater than All Influenza when participants were co-infected with both Influenza A and Influenza B. All Influenza hazard ratios were calculated using the participants’ first infection with influenza.

^b^ The denominator is a sum of individuals enrolled each year. The fact that an individual could contribute to more than one year was taken into account in the analysis using a nested frailty model. In the nested frailty model, individuals are nested within colonies. To maintain the nested data structure, individuals that changed colony during the study periods were excluded from the pooled analysis. There were 32 participants changed their colony during the study period. Of these, 28 cases were originally from five influenza vaccinated colonies and four cases from three hepatitis A vaccinated colonies. One individual (31 years old) was infected by influenza A H3N2 in season 1 and one individual (38 years old) was infected by influenza B in season 3. They both were from influenza vaccinated colonies.

^c^ A robust sandwich variance estimator was used with Cox proportional hazards to adjust for membership in the randomized colony.

^d^ 15 cases of 2009 H1N1 were detected from May 22 to June 4,2009 in the study population. All these 15 cases were in influenza vaccinated group and excluded from the analysis.

In season 2, we detected 54 laboratory-confirmed influenza cases in the entire study population (Table 3): 30 (1.5%) in the colonies assigned to influenza immunization and 24 (1.3%) in colonies assigned to hepatitis A, and all of them were 2009 H1N1. Of the 54 cases, 29 were nonrecipients (Table 2): 18 (1.2%) in the colonies assigned to influenza immunization and 11 (0.9%) in colonies assigned to hepatitis A. The vaccine had no indirect protective effectiveness: -38% (95% CI, -574% to 72%; P = 0.69).The vaccine also had no protective effectiveness among all study participants: -11% (95% CI, -447% to 78%; P = 0.90).

In season 3, laboratory-confirmed influenza was detected in 70 nonrecipients (Table 3): 29 (1.8%) in the colonies assigned to influenza vaccine group (7 H3N2, and 22 influenza B by RT-PCR) and 41 (3.3%; 34 H3N2, 2 unknown influenza A, and 5 influenza B by PCR) in colonies assigned to hepatitis A. The level of indirect vaccine protective effectiveness among nonrecipients was 44% (95% CI, -56% to 80%; P = 0.27) (Table 3). The level of vaccine protective effectiveness among all study participants was 52% (95% CI, -32% to 83%; P = 0.16) (Table 4).

The pooled analysis through a nested frailty model that combined Season 1 and Season 3 (Season 2 was excluded due to the pandemic in this season) showed that the indirect protective effectiveness among nonrecipients was 60% (95% CI, 6% to 83%; P = 0.04) (Table 3). The protective effectiveness among all study participants was 63% (95% CI, 6% to 83%; P = 0.04) (Table 4). In order to maintain the nested data structure, the 32 individuals who changed colony during the study periods were excluded from the pooled analysis. Of these, 28 cases were originally from five influenza vaccinated colonies and four cases from three hepatitis A vaccinated colonies. One individual (31 years old) was infected by influenza A H3N2 in season 1 and one individual (38 years old) was infected by influenza B in season 3; both were from influenza vaccinated colonies.

Further analysis suggested that the indirect protectiveness among nonrecipients varied for influenza A compared to influenza B. For influenza A, the indirect protective effectiveness for Season 1 and Season 3 combined was 77% (95% CI, 25% to 93%; P = 0.02) (Table 3). For influenza B, the protective effectiveness for Season 1 and Season 3 combined was -5% (95% CI, -272% to 71%; P = 0.95) (Table 3).

A similar pattern of difference was found among all study participants in the protectiveness against influenza A and influenza B. For influenza A, the protective effectiveness for Season 1 and Season 3 combined was 82% (95% CI, 43% to 95%; P = 0.00) (Table 4). For influenza B, the protective effectiveness was -28% (95% CI, -480% to 72%; P = 0.75) (Table 4).

This difference was also confirmed by the single season analyses in both seasons 1 and 3. In Season 1, for influenza A, the protective effectiveness was 69% (95% CI, -4% to 91%; P = 0.06) among nonrecipients (Table 3) and was 74% (95% CI, 4% to 93%; P = 0.04) among all study participants (Table 4). For influenza B, the protective effectiveness was 34% (95% CI, -149% to 83%; P = 0.57) among nonrecipients (Table 3) and was 33% (95% CI, -169% to 83%; P = 0.54) among all study participants (Table 4).

In Season 3, for influenza A, the protective effectiveness was 85% (95% CI, 48% to 96%; P < 0.01) among nonrecipients (Table 3) and was 89% (95% CI, 63% to 97%; P < 0.01) among all study participants (Table 4). For influenza B, the protective effectiveness was -150% (95% CI, -828% to 33%; P = 0.17) among nonrecipients (Table 3) and -86% (95% CI, -748% to 59%; P = 0.42) among all study participants (Table 4).

#### Secondary outcome

In a pooled analysis of season 1 and 3 combined, comparing the influenza group to the hepatitis A group in all participants, we found odds ratios for influenza-like illness of 0.57 (95%CI: 0.33 to 0.99, P = 0.05), antimicrobial prescriptions 0.66 (95%CI: 0.43 to 1.01, P = 0.06), medically attended visits for respiratory illness 0.69 (95%CI: 0.47 to 1.03, P = 0.07), emergency department visits, 1.04 (95%CI: 0.33 to 3.3, P = 0.95), and school or work related absenteeism 0.65 (95%CI: 0.41 to 1.04, P = 0.07) (Table 5).

**Table 5:** Association of secondary outcomes in all participants ^a^.

	Influenza Vaccine Colonies	Hepatitis A Vaccine Colonies	Odds Ratio (95%CI)	P value
	No (%)	No (%)		
I**nfluenza-like illness**				
**All Seasons**	133/5922 (2.2%)	166/5063 (3.3%)	0.7 (0.43 to 1.12)	0.14
**Season 1 &3**	97/3876 (2.5%)	144/3251 (4.4%)	0.57 (0.33 to 0.99)	0.05
Season 1(2008–2009)	60/1773 (3.4%)	87/1500 (5.8%)	0.59 (0.28 to 1.22)	0.15
Season 2(2009–2010)	36/2046 (1.8%)	22/1812 (1.2%)	1.5 (0.64 to 3.53)	0.35
Season 3 (2010–2011)	37/2103 (1.8%)	57/1751 (3.3%)	0.57 (0.25 to 1.27)	0.17
**Antimicrobial prescriptions**				
**All Seasons**	213/5922 (3.6%)	263/5063 (5.2%)	0.69 (0.49 to 0.98)	0.04
**Season 1 &3**	158/3876 (4.1%)	197/3251 (6.1%)	0.66 (0.43 to 1.01)	0.06
Season 1(2008–2009)	89/1773 (5%)	122/1500 (8.1%)	0.67 (0.39 to 1.15)	0.14
Season 2(2009–2010)	55/2046 (2.7%)	66/1812 (3.6%)	0.76 (0.43 to 1.36)	0.36
Season 3 (2010–2011)	69/2103 (3.3%)	75/1751 (4.3%)	0.67 (0.36 to 1.25)	0.21
**Medically attended visit for respiratory illness**				
**All Seasons**	282/5922 (4.8%)	312/5063 (6.2%)	0.77 (0.55 to 1.08)	0.13
**Season 1 &3**	207/3876 (5.3%)	242/3251 (7.4%)	0.69 (0.47 to 1.03)	0.07
Season 1(2008–2009)	110/1773 (6.2%)	140/1500 (9.3%)	0.7 (0.42 to 1.19)	0.19
Season 2(2009–2010)	75/2046 (3.7%)	70/1812 (3.9%)	1.01 (0.57 to 1.8)	0.97
Season 3 (2010–2011)	97/2103 (4.6%)	102/1751 (5.8%)	0.69 (0.38 to 1.24)	0.22
**Emergency department visits**				
**All Seasons**	25/5922 (0.4%)	24/5063 (0.5%)	0.85 (0.3 to 2.4)	0.76
**Season 1 &3**	24/3876 (0.6%)	18/3251 (0.6%)	1.04 (0.33 to 3.3)	0.95
Season 1(2008–2009)	22/1773 (1.2%)	16/1500 (1.1%)	1.18 (0.35 to 4.02)	0.79
Season 2(2009–2010)	1/2046 (0%)	6/1812 (0.3%)	0.16 (0.02 to 1.44)	0.10
Season 3 (2010–2011)	2/2103 (0.1%)	2/1751 (0.1%)	0.85 (0.13 to 5.71)	0.86
**School or work related absenteeism**				
**All Seasons**	289/5922 (4.9%)	344/5063 (6.8%)	0.68 (0.43 to 1.06)	0.09
**Season 1 &3**	255/3876 (6.6%)	301/3251 (9.3%)	0.65 (0.41 to 1.04)	0.07
Season 1(2008–2009)	85/1773 (4.8%)	114/1500 (7.6%)	0.62 (0.29 to 1.31)	0.21
Season 2(2009–2010)	34/2046 (1.7%)	43/1812 (2.4%)	0.73 (0.31 to 1.75)	0.48
Season 3 (2010–2011)	170/2103 (8.1%)	187/1751 (10.7%)	0.65 (0.36 to 1.16)	0.15

^a^ Generalized estimating equations were used to adjust for membership in the randomized clusters with the logit-link function.

In a pooled analysis of the three seasons, comparing the influenza group to the hepatitis A group in all participants, we found odds ratios for influenza-like illness of 0.70 (95%CI: 0.43 to 1.12, P = 0.14), antimicrobial prescriptions 0.69(95%CI: 0.49 to 0.98, P = 0.04), medically attended visits for respiratory illness 0.77(95%CI: 0.55 to 1.08, P = 0.13), emergency department visits, 0.85 (95%CI: 0.3 to 2.4, P = 0.76), and school or work related absenteeism 0.68 (95%CI: 0.43 to 1.06, P = 0.09) (Table 5).

There were no significant differences in these outcomes between groups in any of the study seasons (Table 5).

## Discussion

The pooled analysis from seasons 1 and 3 of this trial demonstrates that immunization of children and adolescents with trivalent inactivated influenza vaccine offers significant herd protection from laboratory-confirmed influenza among vaccine nonrecipients; moreover the effect sizes were similar for both seasons among both nonrecipients and the entire study population (61% vs. 44%; 59% vs. 52% respectively).

Further analysis for influenza A and influenza B for pooled data of seasons 1 and 3 suggests that this protection was effective for influenza A, but not influenza B due to the differences in lineage between the influenza B vaccine and the circulating strains [17, 19]. The indirect protective effectiveness estimates for influenza A among nonrecipients and among all participants were 77% and 82% respectively.

The single season analysis in Season 3 suggested that immunizing children with inactivated influenza vaccine did not have a statistically significant effect among either nonrecipients or all study participants, although these results were consistent with Season 1 in direction (44% vs. 61%, and 52% vs. 59% respectively). However, the results of our pooled analysis of Season 1 and 3 show the protection was statistically significant. The most likely explanation is the matching for influenza A and differences in lineage between influenza B antigens in the vaccine and circulating strains. For influenza A, the recommended vaccines were A/California/7/2009[H1N1]–like virus and A/Perth/16/2009[H3N2]–like virus, which matched with the circulating strains [19]. For influenza B, the recommended vaccine was B/Brisbane/60/2008 –like virus, while the circulating strains included B/Brisbane/60/2008 –like virus and B/Wisconsin/01/2010-like virus [19]. For influenza A there was a good match between the recommended vaccines and circulating virus, and the results were significant in both nonrecipients (p<0.01) and in all participants (p<0.01); while, the results were not significant for influenza B.

The results presented above continue to show that the effects of the indirect protection of immunizing children and adolescents on study participants was substantial, especially when there was a good match between the vaccine and the circulating influenza strain. The substantial indirect protectiveness further offer scientific evidence about the ability of inducing herd immunity through vaccination.

Immunizing children in Hutterite colonies provides a unique opportunity to assess herd protection with inactivated influenza vaccines. In contrast to other observational studies [7, 9, 12, 13] and randomized trials [8, 10, 11, 25–27], the contact networks characterized within Hutterite communities allows for the direct assessment of child-to-adult transmission and prevents the reintroduction of novel influenza strains. In addition, our trial extended over three seasonal periods, in order to account for the variation in influenza outbreaks and to determine consistency of effect. Through this trial, we were able to show that vaccinating children and adolescents with trivalent inactivated influenza vaccine could consistently provide unimmunized community members with a significant herd protection from laboratory-confirmed influenza.

We acknowledge the limitations in our study. First, the unique nature of the Hutterite communities may limit extrapolation of our findings to other communities. However, Hutterite communities do share some characteristics of other communities. For example, Hutterites are made up of single families each living in their own house; Hutterites shop in nearby towns for supplies and clothing not available on their colonies; Hutterite children attend school; and Hutterite families regularly socialize with friends and relatives. Although influenza transmission networks in Hutterite communities may be different from that in other communities, there are no data showing this with assurance. Second, we also acknowledge that the number of cases for subtypes of influenza A and influenza B are small with resulting wide confidence intervals. However, we did not power the study for these outcomes. The sample size was derived for influenza A and B together. Third, children aged 24 to 36 months did not receive the study vaccine. The effect size may have been greater had younger children been vaccinated along with those aged 3 to 15 years.

In conclusion, this report demonstrates further evidence of a community-based herd effect as a result of immunizing children against seasonal influenza. This vaccination strategy has the potential to provide a cost-effective method of reducing the burden of influenza-.

## Supporting Information

S1 Data (XLSX)

S1 Fig CONSORT Flow Diagram. (DOC)

S1 Table Direct Vaccine Protective Effectiveness. (DOC)

S1 Text Research Protocol. (DOC)

S2 Text CONSORT Checklist. (DOC)
