# Supplementary material for: Effect of Influenza Vaccination of Children on Infection Rate in Hutterite Communities: Follow-Up Study of a Randomized Trial
Source: PLoS One. 2016 Dec 15;11(12):e0167281. doi: 10.1371/journal.pone.0167281 (PMC5157992; doi:10.1371/journal.pone.0167281)
Supplement: S1 Fig — (DOC) [file pone.0167281.s002.doc]

**
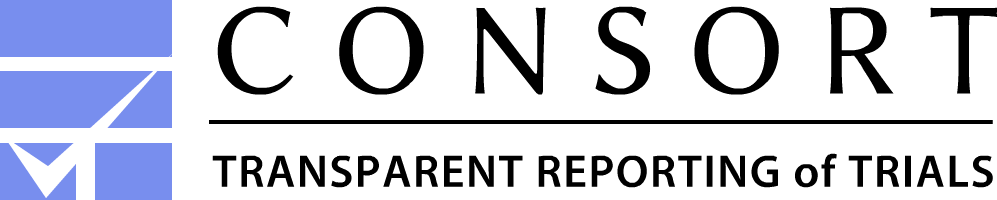
**

**CONSORT 2010 Flow Diagram**

**Allocation**

**Analysis**

**Follow-Up**

**Enrollment**

Colonies Assessed for eligibility (n= 248)

Colonies Excluded (n= 180)

♦  Not meeting inclusion criteria (n=27)

♦  Declined to participate (n=153 )

Analysed (n=5922 person-season)

Colonies allocated to influenza group (n= 35)

Colonies allocated to Hepatitis (n=33)

Analysed (n=5063 person-season)

Colonies Randomized (n=68)

**All Three Study Seasons**

Vaccinated Children (n=1554 person-season)

Non-vaccine recipients (n=4368 person-season)

Vaccinated Children (n=1460 person-season)

Non-vaccine recipients (n=3603 person-season)

**2008-2009 Flu Season**

**Allocation**

**Analysis**

**Follow-Up**

Analysed (n=1773)

Vaccinated Children (n=502)

Non-vaccine recipients (n=1271)

Colonies allocated to influenza group (n= 25)

♦ Received allocated intervention (n=22 )

♦ Did not receive allocated intervention (withdraw) (n=3)

Vaccinated Children (n=445)

Non-vaccine recipients (n=1055)

Colonies allocated to Hepatitis (n=24)

♦ Received allocated intervention (n=24 )

Analysed (n=1500)

Colonies Randomized (n=49)

**2009-2010 Flu Season**

**Allocation**

**Analysis**

**Follow-Up**

Analysed (n=2046)

Vaccinated Children (n=525)

Non-vaccine recipients (n=1521)

Colonies allocated to influenza group (n= 29)

♦ 1 colony from 2008-2009 season discontinued (209 participants)

♦ 8 new colonies added (482 participants)

Vaccinated Children (n=528)

Non-vaccine recipients (n=1284)

Colonies allocated to Hepatitis (n=30)

♦ 2 colonies from 2008-2009 season discontinued (246 participants)

♦ 8 new colonies added (558 participants)

Analysed (n=1812)

Colonies Randomized (n=59)

**2010-2011 Flu Season**

**Allocation**

**Analysis**

**Follow-Up**

Analysed (n=2103)

Vaccinated Children (n=527)

Non-vaccine recipients (n=1576)

Colonies allocated to influenza group (n= 30)

♦ 2 colonies from 2009-2010 season discontinued (157 participants)

♦ 2 new colonies added (241 participants)

Vaccinated Children (n=487)

Non-vaccine recipients (n=1264)

Colonies allocated to Hepatitis (n=28)

♦ 2 colonies from 2009-2010 season discontinued (249 participants)

♦ 1 new colony added (188 participants)

Analysed (n=1715)

Colonies Randomized (n=58)
