# Supplementary material for: Effect of Influenza Vaccination of Children on Infection Rate in Hutterite Communities: Follow-Up Study of a Randomized Trial
Source: PLoS One. 2016 Dec 15;11(12):e0167281. doi: 10.1371/journal.pone.0167281 (PMC5157992; doi:10.1371/journal.pone.0167281)
Supplement: S1 Table — (DOC) [file pone.0167281.s003.doc]

**Supplement Table**: Protective effectiveness for vaccinated group

|  | **Influenza Vaccine Colonies** | **Hepatitis A Vaccine Colonies** | **Hazard Ratio (95% CI)** | **Protective Effectiveness (95%CI)** | **P value** |
| --- | --- | --- | --- | --- | --- |
| RT-PCR -confirmed Influenza | | | |  |  |
|  |  |  |  |  |  |
| **Primary Outcome** |  |  |  |  |  |
| All Influenza |  |  |  |  |  |
| **All Seasons** | 71/1527 ( 4.6%) | 131/1459( 9%) | 0.44 ( 0.17 to 1.14 ) | 56 ( -14 to 83 ) | 0.0900 |
| **Season 1&3** | 59/1009 ( 5.8%) | 118/931( 12.7%) | 0.44 ( 0.16 to 1.2 ) | 56 ( -20 to 84 ) | 0.1100 |
| Season 1 (2008-2009) | 41/502( 8.2%) | 79/445( 17.8 %) | 0.45 ( 0.17 to 1.24 ) | 55 ( -21 to 84 ) | 0.1120 |
| Season 2 (2009-2010) | 12/525( 2.3%) | 13/528( 2.5 %) | 0.93 ( 0.13 to 6.45 ) | 7 ( -545 to 87 ) | 0.9410 |
| Season 3 (2010-2011) | 18/527( 3.4%) | 39/487( 8 %) | 0.41 ( 0.13 to 1.29 ) | 59 ( -29 to 87 ) | 0.1290 |
| **Secondary Outcomes** |  |  |  |  |  |
| Influenza A |  |  |  |  |  |
| **All Seasons** | 22/1527 ( 1.4%) | 79/1459( 5.4%) | 0.22 ( 0.07 to 0.69 ) | 78 ( 31 to 93 ) | 0.0092 |
| **Season 1&3** | 10/1009 ( 1%) | 66/931( 7.1%) | 0.14 ( 0.03 to 0.66 ) | 86 ( 34 to 97 ) | 0.0120 |
| Season 1 (2008-2009) | 8/502( 1.6%) | 37/445( 8.3 %) | 0.19 ( 0.04 to 0.97 ) | 81 ( 3 to 96 ) | 0.0452 |
| Season 2 (2009-2010) | 12/525( 2.3%) | 13/528( 2.5 %) | 0.93 ( 0.13 to 6.45 ) | 7 ( -545 to 87 ) | 0.9410 |
| Season 3 (2010-2011) | 2/527( 0.4%) | 29/487( 6 %) | 0.06 ( 0.01 to 0.32 ) | 94 ( 68 to 99 ) | 0.0009 |
| Influenza B |  |  |  |  |  |
| **All Seasons** | 49/1527 ( 3.2%) | 52/1459( 3.6%) | 1.5 ( 0.31 to 7.31 ) | -50 ( -631 to 69 ) | 0.6200 |
| **Season 1&3** | 49/1009 ( 4.9%) | 52/931( 5.6%) | 1.45 ( 0.31 to 6.69 ) | -45 ( -569 to 69 ) | 0.6300 |
| Season 1 (2008-2009) | 33/502( 6.7%) | 42/445( 9.4 %) | 0.72 ( 0.17 to 3.12 ) | 28 ( -212 to 83 ) | 0.6600 |
| Season 2 (2009-2010) | NA | NA | NA | NA |  |
| Season 3 (2010-2011) | 16/527( 3%) | 10/487( 2.1 %) | 1.48 ( 0.26 to 8.4 ) | -48 ( -740 to 74 ) | 0.6580 |
