# Supplementary material for: Effect of Influenza Vaccination of Children on Infection Rate in Hutterite Communities: Follow-Up Study of a Randomized Trial
Source: PLoS One. 2016 Dec 15;11(12):e0167281. doi: 10.1371/journal.pone.0167281 (PMC5157992; doi:10.1371/journal.pone.0167281)
Supplement: S1 Text — (DOC) [file pone.0167281.s004.doc]

**Research proposal**

**1.0 THE NEED FOR A TRIAL**

**1.1 What is the problem to be addressed?** Despite immunization of persons at high risk for complications of influenza, annual epidemics continue to pose an important threat to the health of persons over the age of 65 years as well as to persons with cardiopulmonary and other high risk conditions. The efficacy of the inactivated influenza vaccine is reduced in these groups due to their inability to mount an adequate immune response. Alternative strategies are therefore needed to better protect those at high risk of complications. Healthy children are felt to be an important source for community transmission of influenza. Immunizing healthy school-aged children, who mount a robust immune response to the vaccine, has been proposed as one strategy to protect high risk individuals. By immunizing children, spread of influenza is prevented and persons at high risk for complications are protected. Although observational data are supportive, a randomized controlled trial is needed to test whether such a strategy provides indirect benefit. Many challenges exist to carefully assessing the indirect benefit of influenza immunization, including the feasibility of extensive laboratory testing in large clusters of high risk persons in towns and cities, the problem of repeated introduction of influenza in a community, and the uncertain uniformity and nature of interactions between healthy children and individuals at high risk for complications of influenza. Hutterites, along with the Mennonites, were founded as Protestant sects in the 16^th^ century Anabaptist movement of Switzerland. The majority of Hutterites live in Alberta, Saskatchewan, and Manitoba where they practice communal farming on small colonies relatively isolated from towns and cities. Randomization of these homogeneous, moderately sized colonies where regular influenza transmission is facilitated by a communal lifestyle, but where there is limited re-introduction of the virus because of isolation from the outside community, represents a unique opportunity to test the hypothesis of indirect benefit.

**1.2 What is/are the principal research question(s) to be addressed?** The goal of this study is to test whether immunizing children in Hutterite colonies with inactivated influenza vaccine can prevent laboratory-confirmed influenza in other community members, including older adults, very young children, and others at high risk for complications. We hypothesize that if ≥70% of healthy children in intervention colonies are immunized with inactivated vaccine, laboratory-confirmed influenza in other Hutterite colony members will be reduced. Specific objectives of the proposed cluster randomized trial are to assess whether high immunization rates among healthy children in Hutterite colonies reduces laboratory-confirmed influenza in 1) other colony members, 2) in high risk participants (including the elderly, individuals with chronic medical conditions, pregnant women, and young children), 3) in the , healthy children immunized, , as well as whether the intervention reduces the following among all participants: influenza-like illness, antimicrobial prescriptions, physician-diagnosed otitis media, school or work-related absenteeism, physician visits for respiratory illness, lower respiratory infection, pneumonia, hospitalizations, and death.

**1.3 Why is a trial needed now? Evidence from the literature.**

**1.3.1 Burden of influenza:** Influenza virus causes annual epidemics of respiratory illness worldwide and is the most important cause of medically attended acute respiratory illness. Influenza and pneumonia together represent the leading infectious cause of death in Canada and the United States and are the sixth leading cause of death overall (1). From 5% to 20% of the population is infected with influenza each year. Approximately 200,000 people in the United States are hospitalized because of influenza-related complications, and 36,000 die as a result of such complications (2, 3). Although influenza affects all age groups, inter-pandemic rates of morbidity are highest in extremes of age, including previously healthy young children aged less than months, adults aged 65 years and older, and other individuals with chronic cardiac, pulmonary, metabolic, and other medical conditions (4, 5).

**1.3.2 Evidence for the role of children in spread of influenza:** Data from a three-year longitudinal surveillance study of children and adults in New York State demonstrated that children were about twice as likely to acquire and shed influenza as compared to adults (6). In a longitudinal study conducted in Seattle from 1968 to 1974, elementary and junior high school students had the highest rates of influenza during epidemics, reaching 54% (7, 8). In the Tecumseh, Michigan studies from 1976 to 1981, over one third of children between the ages of 5 and 14 years had influenza virus isolated from specimens, the highest rate was among persons with febrile respiratory illness (9). In a 2001 surveillance study of 216 families in the Seattle area, for every 100 children followed, influenza accounted for an estimated 20 excess days of work missed by the parents due to illness, demonstrating the potential importance of children in household transmission (10). Although delays in adults seeking medical consultation or the potential for parents over reporting illness in their children (6-8) may have led to a relative underestimation of influenza in adults, the studies nevertheless suggest that children play an important role in community transmission of influenza. We anticipate that the proposed randomized clinical trial will provide rigorous evidence for this role.

**1.3.3 Current vaccination strategy:** With the exception of the province of Ontario, where inactivated influenza vaccine is publicly funded for persons of all ages, current influenza immunization strategy in Canada, the United States, and other developed countries is to immunize persons at high risk of complications (11, 12). This includes people 65 years and older; children 23 months of age or less; adults and children 6 months and older with chronic heart or lung conditions, metabolic diseases, chronic kidney disease, or compromised immune system; children 6 months to 18 years of age on long-term aspirin therapy; women who will be pregnant during the influenza season; and individuals with conditions that can compromise respiratory function. In the United States, children between the ages of 24 months and five years are now considered high risk (12), although rates of hospitalization for this group are substantially lower than in those 23 months of age or less (13, 14).

**1.3.4 Evidence for the potential benefit of immunizing healthy school children:** Given that school-aged children appear to play an important role in introducing and transmitting influenza into households, and hence into the community, immunizing them may interrupt the spread. Monto and colleagues demonstrated that selectively immunizing 86% of children in a 7,500 person Michigan community reduced influenza in adults by a third when compared to an adjacent community where children were not immunized (15). Japan began a program of immunizing school-aged children in 1962 and continued this policy until 1994 (16) (Figure 1). The effect of this policy was to dramatically reduce excess mortality rates to values similar to those in the United States. The fact that there was a rapid increase in excess deaths after 1994, the year in which mass immunization formally ended, supports the conclusion that the effects observed in earlier years were due to vaccine-induced herd immunity (16). Two small trials of inactivated influenza vaccine in children showed a reduction in respiratory illness in unvaccinated household contacts (17, 18). Another study compared the efficacy of live attenuated and inactivated influenza vaccines in Russian schoolchildren aged between seven and 14 years (19). An analysis of the relationship between respiratory illness of staff or unvaccinated children and percentage of children vaccinated in schools in this study showed an inverse relation for live attenuated vaccine but not inactivated vaccine. This unexpected finding, based on data from a single year, is in contrast to the findings of the Tecumseh study and the Japanese experience. This may be because the percentage of children vaccinated in the Russian schools was highly variable, ranging from 23% to 95%, as suggested by the authors. An unblinded, non-randomized trial of immunizing school aged children with a cold adapted, live intra-nasal vaccine demonstrated a reduction in physician visits for acute respiratory illness among adults (20). A recent study compared rates of influenza-like illness among two groups: households with school children offered immunization with live attenuated influenza vaccine and households with school children who had not been offered the vaccine (21). Immunization of 47% of eligible children in the vaccine group was associated with an 11% reduction in influenza-like illness. **1.3.5 Limitations of existing studies:** No randomized controlled trials of the indirect benefit of inactivated vaccines to the community have been reported. Although the studies from Michigan (15) and Japan (16) provide important data demonstrating the potential benefit of immunizing school aged children with inactivated vaccine, these studies are subject to biases associated with observational studies. The school-based immunization study using live attenuated vaccine was only partially randomized because school administrators of four of the 11 school clusters declined to possibly be in a control group where there would be no benefit offered to pupils (21). Inferences that can be drawn about the relative indirect benefits of influenza immunization in the Russian trial are limited since an analysis of indirect benefit was not presented (19). However, observational data analysis derived from this trial along with data from other studies (15, 16) suggest that a high degree of vaccine uptake is required to demonstrate the indirect benefit of inactivated vaccine. Lack of comprehensive laboratory-based detection of influenza is another limitation. To our knowledge, there has not been a clinical trial testing indirect benefit that has included nucleic acid testing, virus isolation, and serology to assess influenza rates. In the recent school-based trial, the outcome was influenza-like illness based solely on a self-reported questionnaire administered to households, without laboratory confirmation (21). As acknowledged by the authors, lack of blinding in this study was a major limitation. Recall or selection bias may explain why the school-based data on absenteeism did not confirm the significant difference between groups found in the household questionnaire data.

immunizing children in daycare with inactivated vaccine demonstrated a reduction of febrile respiratory illness by 42% in unvaccinated household contacts (15). More recently, an open labelled, non-randomized community-based trial of immunizing children from the age of 18 months to 18 years with a cold adapted, trivalent, live intra-nasal vaccine demonstrated a reduction in physician visits for acute respiratory illness among adults (16). Vaccinating approximately 20 to 25% of children were vaccinated reduced respiratory illness of between 10 and 20%. A recent pilot study where children in one school were vaccinated and those in two other schools served as controls found over a 50% reduction in adult medical visits but no difference in emergency visits (17).

**1.3.6 Rationale for studying Hutterite colonies.**

**1.3.6.1 Internal validity:** The unique nature of the Hutterite population offers an important opportunity to understand whether there is an indirect benefit to immunizing children with inactivated influenza vaccine. Hutterite colonies, where members live communally on colonies of typically 80 to 120 people, are relatively isolated from towns and cities. Regular contact with outside community members is limited. However, outbreaks of influenza in Hutterite colonies occur regularly with influenza being introduced from exposure to outsiders. At the age of 36 months children begin school in a single classroom on the colony and continue until the age of 15, at which point they are considered adults. Meals are shared communally, although children and adults eat separately. Importantly, children have regular contact with those adults and others who are at high risk for complications of influenza. Thus, there is a greater likelihood of child to adult influenza transmission and less opportunity for reintroduction of influenza from non-Hutterite communities. Since the colonies are not very large, it is possible to obtain detailed demographic, health, and immunization information for all members. We recognize that there is uncertainty about the exact proportion of children that need to be immunized in order to show indirect benefit. Nevertheless, it has been estimated through modeling that for influenza strains that transmit well among children, immunization of about 70% of children may greatly reduce transmission in entire communities (22- 25). Modeling also suggests that even lower rates of vaccination may yield benefit, such that vaccinating 20% of children could reduce cases by 46% and 80% coverage would reduce rates by 90% (25). Moreover, such estimates may be conservative for the Hutterite community which has stronger contact networks. Hutterite colonies are relatively homogeneous in terms of their genetic lineage and age and sex distributions, which is of benefit for this proposed trial since it reduces the possibility of imbalances between study arms.

**1.3.6.2 External validity:** We acknowledge that the unique nature of the population may limit extrapolation of findings to other populations. However, lack of indirect benefit of inactivated vaccine in such an ideal setting would make it questionable in other settings. On the other hand, an important indirect benefit in such a “natural community laboratory” would confirm observational studies and offer the most rigorous scientific proof available about the ability of inactivated vaccine to induce herd immunity in a manner that cannot feasibly be demonstrated in typical non-Hutterite communities. In fact, we explored the possibility of randomizing small towns with schools in rural Saskatchewan but it soon became clear that this approach would never be feasible because of the relatively large size of the communities. It should be noted that Hutterite colonies share some key characteristics with typical non-Hutterite communities. They are made up of single families each residing in their own house. Hutterite men each have their own occupation, working outside the home, while women spend the majority of their time working in the home. Hutterite families shop in nearby towns for supplies and clothing not available on the colony. The children attend school and Hutterite families regularly socialize with friends and relatives. Although compared to non-Hutterite communities, Hutterite colonies have a higher percentage of young people (34% vs. 17% aged ≤14 years) and larger families (mean 4.6 vs. 3.3 members); the percentage of people with high risk conditions is similar (8% vs. 8.3% for asthma and 6% vs. 4% for diabetes) as is uptake of influenza immunization for those with ≥ 1 chronic condition (40% versus 42%). Generalizing true community-wide indirect benefit to non-Hutterite communities from the proposed trial offers clear advantages over extrapolations limited to household contacts (17,18) or non-randomized two-community comparisons (15, 20).

**1.3.7 Preliminary studies:** Using the same cluster randomized trial design described below, we conducted a pilot study to assess feasibility issues around the main trial. Seven Dariusleut Hutterite colonies in the David Thompson Region of Alberta (Figure 2) were randomized to either the intervention (immunization of healthy children between the ages of 36 months and 15 years with inactivated influenza vaccine) or to the comparison arm, where the children were immunized with hepatitis A vaccine. Beginning February 15, 2006, 178 healthy children were enrolled over 4 weeks. The average number of healthy children enrolled per colony was 25 (range 18 to 33, median 25) representing on average 80% (range 69% to 96%) of children per colony. We also enrolled 60 high risk participants, representing 87% of potentially eligible high risk participants defined by age ≥65 or by chronic medical condition. Because of a late start, baseline specimens could only be obtained from March 24 to April 29, convalescent specimens from June 13 to June 15. Complete sets of specimens were obtained for 57 of the 60 persons enrolled. Two healthy children had influenza detected by PCR and viral culture. Two high risk participants had > four-fold rise in titres to A Wisconsin/H3N2, a non-vaccine strain that circulated that season and one participant who had not been vaccinated had a 64-fold decline in titres to this strain. Three other high risk participants had greater than four fold decline in titres to A Wisconsin/H3N2 (3) and two to B/Malaysia. Although we consider the two seroconverters as definite cases and the third with a high decline in titres with no explanation as a probable case, the other five are possible cases. Based on these data, serological attack rates ranging from 5% (3 of 57) to 14% (8 of 57) are plausible. Since this pilot study only included clinical surveillance following the peak period of influenza activity (from January 8 to March 11), these figures underestimate influenza activity in Hutterite colonies. The pilot study was essential in demonstrating the feasibility of all aspects of the proposed trial, including participant enrollment, shipment and receipt of study vaccine at regional vaccine storage depots, randomization, allocation concealment, and blinding of colonies, transportation of vaccine to Hutterite colonies, vaccination of children on site at the Hutterite colonies, feasibility of hiring and training nurses who live close to the colonies, twice-weekly visits by nurses to each colony, use of the data collection forms, use of self-reported questionnaires to identify symptomatic participants, procurement and transportation of specimens from colonies through local laboratories to the public health laboratory, logistics of obtaining specimens for serology, and the central coordination of these activities by McMaster.

**1.3.7.2 Demographic surveys:** We received peer reviewed funding by the U.S National Institute of Allergy and Infectious Diseases to develop the protocol for this trial. Since we are now requesting funding to conduct the trial, there is no overlap with previous funding received. We have developed a detailed 294-page manual of operations (MOP) for this (http:/www.hutteriteflutrial.ca/). As part of the protocol development, from July 2006 to January 2007, we identified colonies interested in participating in the trial. We obtained the demographic characteristics of potential participants in these colonies through town hall meetings, telephone interviews with Hutterite leaders, and site visits to colonies. We identified Hutterites from 72 colonies who meet criteria for being at high risk of complications from influenza and we identified healthy children eligible for immunization (Table 1). We evaluated 138 colonies, of which 36 were ineligible. Of 102 potentially eligible colonies, 72 (71%) were interested in participating (Figure 3). We learnt that healthy children on study colonies had not been previously immunized against influenza; there are, on average, 15.5 high risk participants per colony; and the average rate of immunization of high risk individuals (on the basis of age and chronic conditions) on study colonies in 2006-2007 was 15% (median 14%).

**1.3.7.3 Laboratory proficiency testing**: To assess the performance of the three provincial laboratories that will be conducting nucleic acid amplification for influenza in this trial, a blinded panel of influenza A (H3N2, H1N1) and B viral lysates was sent to each laboratory on December 22, 2006 (prepared by Dr. Astrid Petrich, McMaster University). Four dilutions of influenza A and B viral lysates in viral transport media were included in the panel with the lowest dilution being at or near the detection limit.  Three replicates for each viral dilution were included for a total of 24 possible influenza A positives and 12 possible influenza B positives.  Negative samples were included in the panel as a single replicate (human metapneumovirus, RSV, and two viral transport media).  The order of samples was randomized; samples were dispensed into 0.5 mL aliquots.  Panels were sent out on dry ice and study laboratories were given approximately 2 weeks to complete testing and send out results.  Diagnostic accuracy was excellent. The sensitivity and specificity of PCR for detection of influenza A was 100% for all three laboratories. For influenza B, sensitivity ranged from 92% to 100%, specificity from 96% to 100%.

**1.4 Give references to any relevant systematic review(s).** There is a recent systematic review (search strategy used for the review was up to January 2004) that included eight clinical trials: six cluster randomized controlled trials (with respect to contact outcomes) assessing the benefit of immunizing school children in family members or siblings, and two observational studies (26). However, the review identified only three observational studies addressing the effect of vaccinating children to protect the wider community. This is the research question being addressed in this proposal; we have described all three studies above (15, 16, 20). The reviewers concluded that “The evidence suggests that vaccinating healthy children against influenza has the potential for reducing the impact of influenza epidemics. However further evidence is needed as limitations of study design or execution mean that the community benefits are difficult to quantify”. **1.5 How will the results of this trial be used?** Findings from the proposed randomized trial will inform policy makers as to whether or not administering inactivated influenza vaccine to children has the potential to lead to indirect benefit in the general population. If the intervention demonstrates a benefit, it will provide experimental proof to support a policy of immunizing healthy children. Studying entire Hutterite communities will inform about the “universal” effect of inactivated vaccine and about the potential cost savings given our proposed economic analysis. On the other hand, lack of indirect benefit of inactivated vaccine in such an ideal setting would make it questionable in other settings. Since the trial will include an economic analysis, estimates of potential cost savings will be provided. The results also have implications for a pandemic vaccine strategy. That is, during a pandemic when there will be a limited amount of vaccine, it may be of advantage to selectively immunize children in order to reduce transmission into the community. Under current policy, children are the last group to be immunized during a pandemic (27). The results will therefore have important implications for vaccine policy.

**1.6 Describe any risks to the safety of participants involved in the trial:** The major risk of influenza or hepatitis A vaccine, although rare (1 per 200,000 doses), is an anaphylactic reaction, characterized by hives, swelling of mouth and throat, difficultly breathing, and low blood pressure. Such a rare event occurs immediately after injection. Less than one third of individuals receiving study vaccine (influenza or hepatitis vaccine) may experience some soreness or redness at the site for 1-2 days. Fever, malaise, nausea, loss of appetite, muscle aches occur infrequently and may last 1-2 days. It is unclear whether **Guillain-Barré s**yndrome is associated with influenza vaccination, however it has been estimated that if there was such an association, it would be at a rate of 1 or 2 incremental episodes of **Guillain-Barré s**yndrome per million doses of influenza vaccine given.

**2.0 THE PROPOSED TRIAL**

**2.1 What is the proposed trial design?** A multi-centre, blinded, cluster randomized controlled trial. Hutterite colonies will be randomized to either vaccination of healthy children with licensed inactivated trivalent influenza vaccine or to vaccination of healthy children with hepatitis A vaccine as a control. The primary outcome is laboratory confirmed influenza among high risk colony members. To control for geographic variation in influenza activity, colonies will be stratified by health region (Figure 3). **2.2 What are the planned trial interventions?**

**2.2.1 Experimental:** Healthy children aged 36 months to 15 years in the intervention colonies will be immunized with inactivated influenza vaccine (VAXIGRIP® vaccine) recommended for the influenza season (, 2008-2009, 2009-2010, or 2010-2011). A 0.5 ml dose of the vaccine will be administered intramuscularly. Previously unvaccinated children who are less than 9 years of age at the time of immunization will receive a second 0.5 ml dose of the influenza vaccine four weeks following the first vaccine as per influenza immunization recommendations.

**2.2.2 Control:** As a control group, healthy children aged 36 months to 15 years will be immunized with AVAXIM ® -Pediatric (hepatitis A vaccine) in a blinded manner such that the schedule of immunization of influenza will be mimicked (Tables 2A and 2B). That is, children previously unvaccinated for influenza who are less than 9 years of age in the comparison colonies will be immunized with AVAXIM-Pediatric. A 0.5 ml dose of the vaccine will be administered intramuscularly. The children will receive a second 0.5ml injection of sterile saline 4 weeks following the first vaccine to maintain blinding for the second influenza vaccine dose. To complete the hepatitis immunization schedule, children will receive a second 0.5 ml dose 12 months following the first vaccine. This will also serve to maintain the blinding for influenza vaccine in the second year. Children ≥ 9 years will receive two doses of hepatitis A vaccine 12 months a part and in the third year will receive saline vaccine. These schedules ensure that children in the control arm of the study will be fully immunized for hepatitis A. **2.2.3 Rationale for choice of control:** The hepatitis A vaccine is well tolerated and will provide a potential health benefit as well as an adequate control for the study. We are aware of at least one recent outbreak of hepatitis A in a Hutterite colony and given the communal lifestyle rapid spread would be expected. An important reason why we selected hepatitis A vaccine as a control (as opposed to saline alone) is that participation in either study arm may potentially confer benefit to participants. Our experience in the pilot study suggests that this was a key factor in willingness of the Hutterites to participate. This also appeals to local public health units who are aware of risks of hepatitis A among Hutterites. Hepatitis A vaccine was used as a control in a CDC influenza vaccine trial in daycare children (17). **2.3 What are the proposed practical arrangements for allocating participants to trial groups?**

**2.3.1 Allocation:** A statistician in the Department of Clinical Epidemiology and Biostatistics at McMaster University who is independent of the study will assign colonies at random within each of eight health regions to one of the two study groups (influenza or hepatitis A), in a 1:1 ratio. For each stratum, random permutations will be generated using a SAS random number generator, with the starting number taken independently for each stratum. The statistician will keep a copy of allocation status of the colonies and forward the codes to Sanofi Pasteur in Toronto where 0.5ml pre-loaded syringes of study vaccine will receive coded study labels to allow for blinding.

**2.3.2 Stratification:** A review of the available data (from 1999-2000 to 2005-2006) shows marked differences in activity between the eight study health regions in the three study provinces (28). Influenza rates within colonies are potentially highly correlated to regional activity. Based on recommendations from experienced virologists in the study public health laboratories familiar with inter and intra-regional patterns and feasibility, we will group study colonies by health region and then randomly allocate colonies within these strata to the intervention or the control group. In addition to controlling for potentially important differences in exposure to influenza, this will also allow us to assess heterogeneity of the effect of the intervention across health regions.

**2.4 What are the proposed methods for protecting against other sources of bias?** To reduce the possibility of selection bias, the allocation of intervention or control status to colonies will occur after ~~high risk~~ participants have been enrolled in the study. This will reduce the possibility for differential consent or recruitment, or differential application of inclusion or exclusion criteria (29). Since the identity of the study vaccines will only be known to a McMaster statistician independent of the trial and to the Scientific Director of Influenza Vaccines at Sanofi Pasteur, the investigators, the study coordinator, vaccinators, outcome assessors, and data and safety monitoring board will remain blind to allocation status. The volume in influenza and hepatitis A study vaccines and saline will be 0.5 ml. Sanofi Pasteur will provide pre-filled syringes with AVAXIM-Pediatric, VAXIGRIP, and saline or diluent. The preloaded syringes will be appropriately coded as study vaccine and masked using study labels. Given that the schedule is dependent on age, there will be two schedules for each study arm (< 9 years and ≥ 9 years) (Tables 2A and 2B). Each pre-filled syringe used in the study will be identified by a numerical code that will include 1) a number from 1 to 60 that represents the colony where it is to be administered, 2) the age category (< 9 years or ≥ 9 years) of children who will receive the vaccine, 3) a dose number reflecting one of the four doses given to children < 9 years over the three year study period or the three doses given to children ≥ 9 years. This method of coding will allow us to track the study vaccines given to children without knowing the identity of the study vaccine since we will be blind to allocation of colony to study group. .

**2.5 What are the planned inclusion/exclusion criteria?**

**2.5.1** **Colonies:** The study population will include Dariusleut Hutterite colonies in Alberta and Saskatchewan as well as Schmiedeleut colonies in Manitoba. Dariusleut and Schmiedeleut Hutterites, although belonging to different sects, are identical with respect to lifestyle and communal living. A colony is eligible if the two following criteria are met:1) The colony is within a 150 kilometer radius of designated cities or towns (Figure 4), 2) The colony has ≥10 members at high risk for complications of influenza. We selected the first criterion because 1) it increases the chance that influenza will be introduced into the Hutterite colony (i.e. the colony is not excessively isolated), 2) based on our pilot study, it is not feasible for research nurses (who live in cities and towns) to travel more than 150 kilometers to reach their first colony, 3) it reduces the number of public health regions involved, facilitating the process of communication as well as developing memoranda of understanding for vaccine depots. The second criterion allows us to assess the effect on high risk participants although the primary analysis will be conducted on all colony members. A colony will be excluded if either one of the following applies: 1) children do not receive any routine vaccinations, 2) local public health policy is to offer influenza immunization to persons beyond a high risk child’s (e.g. child with cystic fibrosis) household. As shown in Figure 3, we have already assessed eligibility criteria of study colonies.

**2.5.2 Participants:** There are two sets of participants in this trial:

**A. Hutterites other than the healthy children who will be immunized**. Although this category as a whole will be used to assess indirect benefit of the vaccine in the main analysis, Hutterites **at high risk for influenza complications** within this category will be assessed in a separate analysis. These are defined as anyone in one or more of the following groups: individuals aged ≥ 65 years, children **23 months of age or less**, **anyone** **with ≥ 1 of the following conditions severe enough to require regular medical follow-up or hospital care: chronic cardiac or pulmonary disorders (including bronchopulmonary dysplasia, cystic fibrosis, and asthma) diabetes mellitus and other metabolic diseases, cancer, immunodeficiency, immunosuppression (due to underlying disease and/or therapy), renal disease, anemia, and hemoglobinopathy, any condition that can compromise respiratory function or the handling of respiratory secretions or that can increase the risk of aspiration.**  **There are no exclusion criteria for this category of participants.**

**B. Healthy children aged 36 months to 15 years who will be immunized as part of the intervention**. Exclusion criteria include: a) anaphylactic reaction to a previous dose of influenza vaccine, b) anaphylactic reaction to hepatitis A vaccine, c) anaphylactic reaction to neomycin, d) **known IgE-mediated hypersensitivity to eggs manifested as hives, swelling of the mouth and throat, difficulty in breathing, hypotension, or shock, e) Guillain-Barré syndrome within eight weeks of a previous influenza vaccine.**

**2.5.3 Immunization of high risk participants:** The public health units have requested that we make it clear to the Hutterites that we are only immunizing healthy children in this trial and to distinguish this from efforts of public health units to immunize high risk individuals. Therefore, we will not immunize high risk participants in this study.

**2.6 What is the proposed duration of treatment period?** The immunizations will be given at the end of October (prior to the influenza season) for each of the three proposed study years. The rationale for repeating the trial over several seasons is to increase the chance that there will be a sufficiently active influenza season to assess the immunization strategy and also to ensure a match between the vaccine strain and a circulating strain. The trial will continue over the three years even if there is a good match the first or second year. This will allow us to determine consistency of the effect.

**2.7 What is the proposed frequency and duration of follow-up?** All participants will be assessed for signs and symptoms of influenza twice weekly over the follow up period. Since each nurse will be traveling to eight colonies (sometimes separated by > 200 kilometers) more frequent visits are generally not feasible. Parents will receive training to administer self nasal swabs when the nurse is unavailable eg symptoms develop on the weekend or bad weather. The follow up period is defined by the start and stop date of the influenza season in each health region. Beginning in late October, the provincial laboratories will provide weekly updates of the number of positive specimens from sentinel sites for each health region. These sentinel towns and cities were selected because they are close to the colonies and have physicians who regularly submit specimens for viral culture. The start date for surveillance for laboratory confirmed influenza in a region will be set as soon as the two following criteria are met: 1) ≥ 1 nasopharygneal or throat swab specimens received from the sentinel sites must be positive for influenza for two consecutive weeks, 2) ≥ two weeks after the last child in the health region’s study colonies has been immunized. **2.8 What are the proposed primary and secondary outcome measures?**

**2.8.1 Primary outcome:** The primary outcome of this study will be laboratory-confirmed influenza infection among Hutterite colony members other than the healthy children immunized as part of the intervention. We will also determine laboratory-confirmed influenza among the immunized healthy children and will assess Hutterites at high risk for complications of influenza in a separate analysis. This primary outcome is the most appropriate because the purpose of the study is to assess whether immunizing children reduces transmission of influenza to other colony members. Signs and symptoms of influenza are non-specific and cannot be differentiated from those of over respiratory viruses (30). Because non-influenza viruses circulate at the same time as influenza, clinical outcomes may be less responsive to the intervention and do not directly address the main study question. **2.8.2 Secondary outcomes:** In addition to laboratory-confirmed influenza, we will measure the following outcomes in all participants: influenza-like illness, courses of antimicrobial prescriptions, physician-diagnosed otitis media, school or work related absenteeism, physician visits for respiratory illness, lower respiratory infection or pneumonia, hospitalization for lower respiratory infection or pneumonia, all cause hospitalizations, deaths due to lower respiratory infection or pneumonia, all-cause deaths.

**2.9 How will the outcome measures be measured at follow up? 2.9.2 Laboratory confirmed influenza:** This will be confirmed on the basis of at least one of the following: detection of viral RNA on the basis of reverse transcriptase PCR, isolation of influenza virus from nasopharyngeal specimens or throat specimens, ≥ four-fold rise in serum antibodies to circulating influenza strain antigens. All nasopharyngeal or throat specimens will be tested for influenza by PCR and those that are positive will be set up for viral culture (Figure 5). All participants will be assessed for signs and symptoms of influenza twice weekly over the follow up period using a (pre-tested) standardized form of self-reported symptoms or signs from study participants or parents. If there are any new symptoms the nurse will contact the participant directly, confirm the symptoms, and obtain nasopharyngeal specimens (or throat swabs for participants ≤ 24 months) if any of one the following symptoms or signs are present: fever (≥ 38° Celcius), cough, nasal congestion, sore throat, headache, sinus problems, muscle aches, fatigue, ear ache or infection, chills. As we did in our pilot study, we will purchase thermometers for participants to ensure that temperature is recorded. **2.9.2.1 PCR:** PCR has been demonstrated to be more sensitive to viral culture alone, and compared to direct immunofluorescence and cell culture assay, fluorogenic reverse transcription PCR was 95% sensitive and 100% specific for detecting influenza (31, 32). Since it does not rely upon the viability of the virus it is a better methodological choice for the detection of cases that would be missed by viral culture four days or more after the acute infection (33-35). Specimens will be submitted to the public health laboratories in the respective province and will be processed in that provincial laboratory. Protocols for submitting specimens have been developed and pilot tested.

**2.9.2.2 Viral culture:** If the reverse transcription PCR test is positive for influenza, the specimen will be set up for viral culture. Twenty percent of viral isolates (selected randomly from colonies by health region) will be submitted to the National Microbiology Laboratory for subtyping. This testing, along with ongoing subtyping of viral isolates in the study provinces, will allow for an accurate determination of circulating influenza strains in Hutterite colonies, allowing us to assess the match between vaccine and circulating strains. **2.9.2.3 Serology:** Although nucleic acid testing is highly sensitive in the acute and early convalescence phase of influenza disease, some participants will either have a mild infection or will be asymptomatic and may not be identified as cases. To identify these cases, serology (haemagglutinin inhibition assay) will be performed (9). Measurement of antibodies to antigens of the two influenza A and the one influenza B vaccine strains will be performed in other group at four time points namely: prior to the influenza season, three weeks following influenza vaccination (for participants that are vaccinated), at mid-season (defined using an epidemiologic curve constructed from regional data provided by the provincial laboratories), and at the end of influenza season (defined when there is no longer influenza cultured for two consecutive weeks in a region). Influenza infection will be defined by detection of ≥ 4-fold increase in influenza-specific haemagglutinin inhibition assay titer between baseline and convalescent serum samples, taking into account the effect of the vaccine. We will obtain sera from all children three weeks following immunization. Once the study is unblinded, we will test the sera in order to ensure that children who received hepatitis A vaccine developed protective titres. Collecting sera in this way will also allow us to document the immunogenicity to inactivated influenza vaccine that children in the intervention colonies received. **2.9.4 Secondary outcomes:** Influenza-like illness will be defined by the presence of fever (≥ 38.0° Celcius) and cough (30). Courses of antimicrobials (name, dose, and duration) will be recorded by the research nurse using prescription information on the medication container or by contacting the prescribing physician if necessary. Otitis media is a common complication of influenza in children and will be ascertained by research nurses through twice weekly monitoring. The diagnosis of otitis media made by the physician will be confirmed by the research nurse (with consent) by contacting the physician’s office. This outcome will be assessed in children aged up to 5 years using a standardized definition (36). School or work related absenteeism (of culturally appropriate tasks), will be directly reported to the nurse during the twice weekly individual assessments. Signs and symptoms of lower respiratory infection and pneumonia (including chest radiograph results) as well as information about deaths will be obtained from the attending physician using standardized definitions (37). Cause of hospitalizations will be assessed by the research nurse through interviews with participants and review of hospital medical records. Deaths will be recorded by the research nurse using medical records. **2.9.5 Adverse reactions:** We will assess for immediate hypersensitivity allergic reactions by having the research nurse monitor participants for 15 minutes following immunization, as is the general practice in public health units in the study provinces. Additionally, each participant that is vaccinated (and their parents) will receive a symptom list to be checked daily for five days following vaccination. Reactogenicity events (i.e. soreness, redness at the site of injection etc.) are common, expected events but nevertheless will be collected and reported as adverse events. All adverse events will be graded for severity (mild to life threatening) and relationship to study product (associated or not associated). **2.10 Will health service research issues be addressed?** We will collect and value health-care resource utilization for participants in both the influenza and hepatitis A immunization study groups. We will take the perspective of the healthcare system for the economic analysis. We will include in the analysis the cost of influenza vaccines, influenza vaccine administration, physician visits, medications (antimicrobials and over the counter medications), hospital admissions, and emergency room visits, costs related to stays in intensive care units and medical wards, participant transport via ambulance, diagnostic imaging and professional fees. Costs for transport and hospitalizations will be obtained from a large hospital participating in a case costing project (38) and professional fees will be derived from a standard provincial professional fee schedules.

**2.11 What is the proposed sample size and what is the justification for the assumptions underlying the power calculations?** In our pilot study 8 of 57 (14%) participants had a greater than four fold rise or decline in titres (see section 1.3.7) due to either a non-vaccine strain or due to a vaccine strain in persons who were not immunized. Although previous studies that have included both viral culture and serology demonstrate that infection rates vary, an estimate of 10%is in keeping with overall community rates for years where either H3N2 or H1N1 predominate (8,9). We will test whether > 70% level of immunization can, over single influenza seasons, reduce the rate of laboratory confirmed influenza infection from 10% to 5%. If the proposed clinical trial was conducted by randomizing individuals and not clusters, 474 participants in each arm would be needed to have 80% power to detect a 50% reduction in influenza (assuming a baseline attack rate of 10%, a risk reduction of 0.50 (5%), , and type 1 error of 0.05). To adjust for the effect of within cluster dependency, a variance inflation factor is calculated using the equation 1 + (m-1)ρ, where m=average size of the cluster (per cluster=50) and ρ= the intracluster correlation coefficient (ICC). ICCs for cluster randomized trial are seldom accurately predicted (39). Furthermore, there is little data on Hutterite colony attack rates as these are not collated by the health regions. Therefore, as recommended (39), we show in Table 3 sensitivity analysis demonstrating the power that can be achieved using a sample size of 60 clusters (the number of colonies needed to address the study question) and the range of ICCs commonly reported in cluster randomized controlled trials. We have assumed a cluster size of 50, which is the typical number of individuals in colonies interested in participating in the study. Table 3 demonstrates that with a sample size of 60 clusters we will be adequately powered to detect a difference over the range of ICCs commonly reported for cluster randomized controlled trials, 0.01 to 0.05. The table also demonstrates that our sample size allows us to assess a 50% reduction in the primary outcome assuming baseline event rates as low as 8% within a range of ICC from 0.01 to 0.04. This equates to a sample size of 1500 participants in each group. For the other comparisons that we plan to assess, sensitivity analysis shows adequate power to assess differences between study groups. For example, for testing the indirect benefit to high risk participants, for 15 high risk participants per colony, enrolling 60 colonies would give 84% power to detect a 50% reduction in laboratory-confirmed influenza (including serology which will increase the baseline event rate) from 12% to 6% between groups assuming an ICC is 0.04 and one sided alpha. The sample size has ≥ 90% power (two sided test, 60 clusters, 50 participants per cluster, ICC of 0.04) to detect the following absolute reductions: 12% to 6% for influenza-like illness, 11% to 5% for antimicrobial prescriptions, 6% to 2% for physician visits, 25% to 16% for children with otitis media, 0.7 versus 0.4 days of absenteeism (22, 40). Rates of hospitalization for participants ≥ 65 years and 6 months to 24 months will be much lower, estimated to be between 1.5 per 1000 individuals per season (13, 14). We acknowledge that for this outcome, along with pneumonia and deaths, the sample size may not be sufficient to detect differences. Nevertheless, we anticipate these data will show trends towards differences and the confidence intervals will help establish whether clinically important effects benefits are plausible with the intervention.

**2.12 What is the planned recruitment rate? How will recruitment be organized?** As documented above, we spent six months assessing 138 Hutterite colonies for eligibility and obtaining detailed demographic information about potential participants (Table 1, Figure 3). Seventy two Hutterite colonies are eligible for the trial and from these we plan to enroll 60. Potential participants on these colonies have also been given information about the trial by nurses who were collecting demographic data (written documentation in the form of question and answer sheets and thorough interviews). Based on our experience in the pilot study, where participants from seven colonies were enrolled by two nurses in four weeks, it will take from four to six weeks for two nurses to enroll participants from seven colonies. Therefore, we anticipate enrollment taking place in a six week period (September to mid-October) prior to the availability of influenza vaccine in each study year. Two research nurses will approach Hutterite families in eight colonies about participation and will obtain written informed consent. Since all of the 8.6 FTE research nurses who participated in the preliminary studies expressed interest in working on the trial if it is funded, this will greatly facilitate enrollment given their familiarity with the colonies and because they have established a relationship with potential participants. **2.13 Are there likely to be any problems with compliance?** As we determined from the pilot study using the same study design as the trial, 80% of eligible children were enrolled and vaccinated and 87% of potentially eligible high risk Hutterites participated. Based on this experience, we anticipate high enrollment from the 60 study colonies that are eligible and interested in participating in the study. To date, it appears as though once a colony has committed to the study, problems with compliance will be minimal. None of the 178 children immunized withdrew from the study. Of the 60 high risk participants enrolled, full serological measurements were obtained from 57 (95%) of the participants. 2.14 What is the likely rate of loss to follow-up? Given that colonies that have allowed us to conduct demographic assessment and surveillance are familiar with the study, it is unlikely that they will withdraw from the study after being randomized. However, if this occurs, because there are 72 colonies eligible and we only need 60, we could enroll other colonies. We do expect that there may be individuals who will not want to or be available to participate in subsequent years. Given that we anticipating enrolling 50 participants per colony, this will not have a major effect on sample size. Because of the nature of the Hutterite upbringing, loss to follow up because of migration is uncommon. Migration from one colony to another is unusual. When colonies reach a large enough size, they split by holding a lottery to decide which families move to the new colony. However, this should not affect the trial since colonies that anticipate splitting over the study period declined to participate in the study. Although there may be healthy children who withdraw from the study, our pilot study has demonstrated a high level of participation such that this should not have a major effect on achieving immunization levels above 70%. Also, in the second and third years of the study, we will enroll new individuals who have become eligible for the high risk group or the healthy child group due to a change in their status (age or diagnosis of a high risk condition). This will offset the effect of individuals who withdraw.

**2.15 How many centres will be involved?** There will be four study sites (University of Calgary, Cypress Health Region, Heartland Health Region, and University of Manitoba) and one coordinating centre (McMaster University). The study will be conducted in 60 Hutterite colonies. Four study laboratories (provincial laboratories for Alberta, Saskatchewan, and Manitoba, and National Microbiology Laboratory) will be involved. These are shown in Figure 6.

**2.16 What is the proposed type of analyses?** We propose assessing whether immunizing children in Hutterite colonies with inactivated influenza vaccine can reduce transmission of laboratory-confirmed influenza to other members of the Hutterite colony. As secondary objectives, we will also assess if the intervention reduces laboratory-confirmed influenza in the high risk participants specifically and also in the immunized children. To compare mean colony differences in laboratory confirmed influenza infection between influenza and hepatitis A groups, we will use an analysis appropriate for cluster-randomized trials, namely, a t-test weighted by an inverse binomial variance weight for binary outcomes (41). We will use a similar approach for other binary outcomes. We will use a t-test weighted by an inverse variance for continuous outcomes, such as days of absenteeism. These weights are proportional to the inverse of the variances of the cluster (Hutterite colony participants) proportions or means. All outcomes will be analyzed on an intention-to-treat basis. We will also determine differences in influenza-like illness and other secondary outcomes between intervention and comparison colonies using weighted t-tests. To avoid lack of independence associated with counting multiple outcomes, each specific outcome in a participant will only be counted once in the analysis for that influenza season. The rationale for repeating the trial over several seasons is to increase the chance that there will be sufficiently active influenza season to assess the immunization strategy and also to ensure a match between the vaccine strain and a circulating strain. The trial will continue over the three years even if there is a good match the first or second year. This will allow us to determine consistency of the effect from season to season. Given annual differences in circulating influenza strains, data from each influenza season must be analysed within the context of whether there is a match or not. A match will be defined when at least one antigen in the vaccine is the same as the predominant influenza isolate that is circulating in the study regions. If there are matches in two or more years but with different antigens, then the results will be analysed separately. The interpretation will be an assessment of the consistency of the intervention i.e is the effect of immunizing children the same in matched years regardless of the vaccine antigen. If there are two or more matched years where the vaccine antigen in those years is the same, then the results will be analysed together. To do this we will conduct a repeated measures analysis of variance. That is, colony rates of influenza will be modeled by intervention group (main effect), colony effect will be nested within the intervention or comparison group, and study year will be included in the model as a covariate. If there are two strains circulating at high or equal levels, this will be taken into account in the analysis with the infections caused by both combined. The primary set of analyses will be to assess the effect of the intervention on laboratory-confirmed influenza. For the secondary outcomes, the analyses will be repeated for each outcome, including rate comparisons. Using multi-level regression modeling, we will use rates of laboratory-confirmed influenza in participants as the dependent variable, and will include random effects for a colony variable and for the health region. Variability in vaccine uptake from colony to colony will be taken into account as a fixed effect. There will be fixed effects for individual immunization and study group. This will be done for all three participant groupings (Hutterites other than healthy children, healthy children, high risk participants. We will conduct similar analyses using secondary outcomes as the dependent variable, and will also include fixed participant level variables including age, sex, and co-morbidities since these may have a prognostic effect on influenza complications. As described above, we will combine analyses in matched years if the antigen is the same but will not combine the analyses if there are matched years but different antigens each year. In terms of interactions, we will explore sex-related differences in infection rates, on the basis of whether women’s role in care for children increases risk for infection. If appropriate (skewed data) we will also conduct the analysis using non-parametric testing.

**2.17 What is the proposed frequency of analyses?** The analyses will be conducted at the end of the three year study. There is no interim analysis planned. The trial will continue over the three years even if there is a good match the first or second year. This will allow us to determine consistency of the effect from season to season. As stated above, pooling results will depend on the match and the antigen.

**2.18 Are there any planned subgroup analyses?** The major subgroup analysis will be to assess the effect of the intervention among high risk participants. It is for this reason that we have selected colonies with 12 or more high individuals. As additional analyses, we plan to assess the effect of the intervention in 1) older adults (> 65 years) and 2) young children (23 months of age or less). **2.19 Has any pilot study been carried out using this design? Yes. See section 1.3.7.**

**3.0 TRIAL MANAGEMENT 3.1 What are the arrangements for day to day management of the trial?** The coordinating center of this clinical trial will be at McMaster University. Site investigators will work with research nurses to ensure complete and thorough data collection. As our experience conducting the pilot study and demographic survey confirmed, the research nurses will need to be in regular close contact with the Coordinating Centre. This allows activities in the field to be monitored on an ongoing basis. As the trial progresses, there will be weekly teleconferences with site investigators and research nurses to review progress and troubleshoot problems. There will be annual meetings to do an in-depth review of trial progress and to begin the planning process for the next year. The Coordinating Centre will submit reports, as necessary, to the DSMB and to the university REBs. The three study sites will work closely with the Coordinating Centre to implement the clinical trial (Figure 6). The detailed timeline and schedule of events and study forms are shown in Tables 4 and 5 respectively. We believe that by far the best approach to missing data is to minimize their occurrence in the first place. Our proposed computer management system, using Teleform, where hard copies of data collection forms are faxed and directly entered into the database, will allow us to identify data that appears inconsistent, incomplete, or inaccurate. Nurses will fax in their data collection forms on a weekly basis and these will be checked immediately. We will perform quarterly audit checks as specified in our protocol. All aspects of trial management are detailed in the manual of operations. **3.2 What will be the role of each principal applicant and co-applicant proposed?** Dr. Loeb, the Principal investigator, has experience conducting large multi-centre studies and cluster randomized controlled trials (42-44). He will have overall responsibility for the scientific and logistic conduct of the clinical trial and will chair the steering committee. Dr. Loeb will work closely with the central study coordinator, investigators, steering committee, Hutterite community, health regions, and Sanofi Pasteur to ensure proper implementation of the clinical trial. Dr. Russell, who has experience conducting multi-centre clinical trials of pediatric and adult vaccines (including influenza) in rural Alberta infants and young children (45-47), will be site investigator for Alberta. Drs. Chokani and Vooght, Medical Officers of Health in Saskatchewan, will be site investigators and will work with the PI in trial implementation. Dr. Aoki, an expert in influenza research (48), will be the site investigator for Manitoba. Drs. Fox, Horsman, and Van Caeseele, Directors of Provincial laboratories, have expertise in virology and molecular diagnostics (49), will be responsible for supervising the nucleic acid amplification and viral culture that will be conducted in their provincial laboratories. Dr. Babiuk, a molecular virologist and immunologist, will help guide the scientific direction of laboratory aspects of the clinical trial (50). Dr. Earn, a mathematical modeler with expertise in transmission dynamics of influenza, will help with decision making processes in trial implementation and analysis (51-53). Dr. Goeree, a health economist with clinical trial expertise (44), will conduct the proposed economic analyses. Dr. Walter, a senior statistician at McMaster, is an expert in design and analysis of clinical trials (54-55), will help address both design and analysis issues. **3.3 Describe the trial steering committee and data safety and monitoring committee. 3.3.1 The trial steering committee:** This committee will be composed of the Principal Investigator, who will chair the committee, the site investigators, and our biostatistician. The steering committee will have responsibility for the overall conduct of the trial and will review changes to the protocol, data collection practices, and essential study documents. The steering committee will also be responsible for reviewing and implementing recommendations from the DSMB. The committee will hold annual meetings to ensure that there have been no major deviations from the protocol. **3.3.2 The Data Safety and Monitoring Board (DSMB):** This committee will be responsible for safety oversight of the study (including monitoring of adverse reactions). The DSMB composed of a pediatrician, an infectious disease physician and a biostatistician will be responsible for making recommendations on safety issues, premature trial termination, and unblinding of study groups. The DSMB, which will be blinded to study group, will be asked to review safety data on an annual basis for each arm of the study. If safety concerns arise, more frequent meetings will be initiated. The DMSB will receive immediate notification and reports of any serious adverse reactions.

**References:**

1. Langley JM, Faughnan ME. Prevention of influenza in the general population. CMAJ 2004;171:1213-1222.
2. Thompson WW, Shay DK, Weintraub E, et al. Influenza-associated hospitalizations in the United States. JAMA. 2004;292 :1333 –1340.
3. Centers for Disease Control and Prevention. Key facts about the flu: how to prevent the flu and what to do if you get sick. www.cdc.gov/flu/keyfacts.htm.
4. Editorial Board Respiratory Disease in Canada. Respiratory disease in Canada. Ottawa: Health Canada;2001.
5. Glezen WP, Keitel WA, Taber LH, Piedra PA, Clover RD, Couch RB. Age distribution of patients with medically-attended illnesses caused by sequential variants of influenza A/H1N1: comparison to age-specific infection rates, 1978–1989. Am J Epidemiol 1991;133(3):296-304.
6. Long CE, Hall CB, Cunningham CK, Weiner LB, Alger KP, Gouveia M, et al. Influenza surveillance in community-dwelling elderly compared with children. Arch Fam Med 1997; 6:459-465.
7. Foy HM, Cooney MK, Allan I. Longitudinal studies of types A and B influenza among Seattle school children and families, 1968-1974, J Infect Dis 1976; 134: 362-369.
8. Fox JP, Hall CE, Cooney MK, Foy HM. Influenza virus infections in Seattle families, 1975-1979. Study design, methods and the occurrence of infections by time and age. Am J Epidemiol 1982; 116:212-227.
9. Monto AS, Koopman JS, Longini IM, Jr. Tecumseh study of illness. XIII. Influenza infection and disease, 1976-1981. Am J Epidemiol 1985;121:811-822.
10. Neuzil KM, Hohlbein C, Zhu Y. **Illness Among Schoolchildren During Influenza Season. Effect on School Absenteeism, Parental Absenteeism From Work, and Secondary Illness in Families.** Arch Pediatr Adolesc Med 2002;156:986-991.
11. Statement on Influenza Vaccination for the 2005-2006 Season. National Advisory Committee on Immunization. Canada Communicable Disease Report Volume 31 • ACS-6 15 June 2005.
12. Prevention and Control of Influenza. Recommendations of the Advisory Committee on Immunization Practises. MMWR 2005; 54 (RR08):1-40.
13. Neuzil KM, Mellen BG, Wright PF,Mitchel EF, Griffen MR. Effect of influenza on hospitalizations, outpatient visits, and courses of antibiotics in children. N Engl J Med 2000; 342:225-31.
14. Izurieta HS, Thompson WW, Kramarz P, et al. Influenza and the rates of hospitalization for respiratory disease among infants and young children. N Engl J Med 2000; 342:232--9.
15. Monto A, Davenport FM, Napier JA, Francis T. Modification of an outbreak of influenza in Tecumseh, Michigan by vaccination of schoolchildren. Jour Infect Dis 1970; 122:16-25.
16. Reichert TA, Sugaya N, Fedson DS, et al. The Japanese experience with vaccinating school children against influenza. N Engl J Med 2001; 344:889-96.
17. Hurwitz ES, Haber M, Chang A, et al. Effectiveness of influenza vaccination of day care children in reducing influenza-related morbidity among household contacts. JAMA 2000; 284:1677-1682.
18. Esposito S, Marchisio P, Cavagna R, et al. Effectiveness of influenza vaccination of children with recureent respiratory tract infections in reducing respiratory-related morbidity within the households. Vaccine 2003; 21:3162-68.
19. Rudenko LG, Slepushkin AN, Monto AS, et al. Efficacy of live attenuated and inactivates influenza vaccines in schoolchildren and their unvaccinated contacts in Novgorod, Russia. J Infect Dis 1993; 168:881-887.
20. Piedra PA, Gaglani MJ, Kozinetz CA, et al. Herd immunity in adults against influenza-like illnesses with use of the trivalent-live attenuated influenza vaccine (CAIV-T) in children. Vaccine 2005; 1540-1548.
21. King, JC Jr., Stoddard JJ, Gaglani MJ, et al. Effectiveness of School-Based Influenza Vaccination. N Engl J Med 2006; 355:2523-32.
22. Longini IM Jr, Koopman JS, Monto AS, Fox JP. Estimating household and community transmission parameters for influenza. Am J Epidemiol 1982; 115:736-751.
23. Longini IM, Ackerman E, Elveback LR. An optimization model for influenza A epidemics. Math Biosci 1978; 38: 141-57.
24. Halloran ME, Longini IM, Cowart DM, et al. Community interventions and the epidemic prevention potential. Vaccine 2002; 20: 3254-62.
25. Weycker D, Edelsberg J, Halloran ME, et al. Population-wide benefits of routine vaccination of children against influenza. Vaccine 2005; 26;23:1284-93.
26. Jordon R, Connock M, Albon E, et al. Universal vaccination of children against influenza: are there indirect benefits to the community? A systematic review of the evidence. Vaccine 2006; 24:1047-1062.
27. The Canadian Pandemic Influenza Plan for the Health Sector. Public Health Agency of Canada. <http://www.phac-aspc.gc.ca/cpip-pclcpi/>
28. FluWatch. Public Health Agency of Canada. <http://www.phac-aspc.gc.ca/fluwatch/index.html>
29. [Puffer S](http://www.ncbi.nlm.nih.gov.libaccess.lib.mcmaster.ca/entrez/query.fcgi?db=pubmed&cmd=Search&itool=pubmed_Abstract&term="Puffer+S"%5BAuthor%5D), [Torgerson D](http://www.ncbi.nlm.nih.gov.libaccess.lib.mcmaster.ca/entrez/query.fcgi?db=pubmed&cmd=Search&itool=pubmed_Abstract&term="Torgerson+D"%5BAuthor%5D), [Watson J](http://www.ncbi.nlm.nih.gov.libaccess.lib.mcmaster.ca/entrez/query.fcgi?db=pubmed&cmd=Search&itool=pubmed_Abstract&term="Watson+J"%5BAuthor%5D). Evidence for risk of bias in cluster randomised trials: review of recent trials published in three general medical journals. BMJ 2003; 327: 785-9.
30. Call SA, Vollenweider MA, Hornung CA, Simel DL, McKinney WP. Does this patient have influenza? JAMA 2005: 293: 987-97.
31. Habib-Bein NF, Beckwith III WH, Mayo D, Landry ML. Comparison of SmartCycler real-time reverse transcription-PCR assay in a public health laboratory with direct immunofluorescence and cell culture assays in a medical center for detection of influenza A virus. J. Clin. Microbiol 2003; 41:3597-3601.
32. Schweiger B, Zadow I, Heckler R, Timm H, Pauli G. Application of a fluorogenic PCR assay for typing and subtyping of influenza viruses in respiratory samples. J. Clin. Microbiol 2000; 38:1552-1558.
33. [Templeton KE](http://www.ncbi.nlm.nih.gov/entrez/query.fcgi?db=pubmed&cmd=Search&itool=pubmed_AbstractPlus&term="Templeton+KE"%5BAuthor%5D), [Scheltinga SA](http://www.ncbi.nlm.nih.gov/entrez/query.fcgi?db=pubmed&cmd=Search&itool=pubmed_AbstractPlus&term="Scheltinga+SA"%5BAuthor%5D), [Beersma MF](http://www.ncbi.nlm.nih.gov/entrez/query.fcgi?db=pubmed&cmd=Search&itool=pubmed_AbstractPlus&term="Beersma+MF"%5BAuthor%5D), [Kroes AC](http://www.ncbi.nlm.nih.gov/entrez/query.fcgi?db=pubmed&cmd=Search&itool=pubmed_AbstractPlus&term="Kroes+AC"%5BAuthor%5D), [Claas EC](http://www.ncbi.nlm.nih.gov/entrez/query.fcgi?db=pubmed&cmd=Search&itool=pubmed_AbstractPlus&term="Claas+EC"%5BAuthor%5D). Rapid and sensitive method using multiplex real-time PCR for diagnosis of infections by influenza A and influenza B viruses, respiratory syncytial virus, and parainfluenza viruses 1, 2, 3, and 4. [J Clin Microbiol](javascript:AL_get(this, 'jour', 'J Clin Microbiol.');) 2004; 42:1564-9.
34. [Zitterkopf NL](http://www.ncbi.nlm.nih.gov/entrez/query.fcgi?db=pubmed&cmd=Search&itool=pubmed_AbstractPlus&term="Zitterkopf+NL"%5BAuthor%5D), [Leekha S](http://www.ncbi.nlm.nih.gov/entrez/query.fcgi?db=pubmed&cmd=Search&itool=pubmed_AbstractPlus&term="Leekha+S"%5BAuthor%5D), [Espy MJ](http://www.ncbi.nlm.nih.gov/entrez/query.fcgi?db=pubmed&cmd=Search&itool=pubmed_AbstractPlus&term="Espy+MJ"%5BAuthor%5D), [Wood CM](http://www.ncbi.nlm.nih.gov/entrez/query.fcgi?db=pubmed&cmd=Search&itool=pubmed_AbstractPlus&term="Wood+CM"%5BAuthor%5D), [Sampathkumar P](http://www.ncbi.nlm.nih.gov/entrez/query.fcgi?db=pubmed&cmd=Search&itool=pubmed_AbstractPlus&term="Sampathkumar+P"%5BAuthor%5D), [Smith TF](http://www.ncbi.nlm.nih.gov/entrez/query.fcgi?db=pubmed&cmd=Search&itool=pubmed_AbstractPlus&term="Smith+TF"%5BAuthor%5D). Relevance of influenza A virus detection by PCR, shell vial assay, and tube cell culture to rapid reporting procedures [J Clin Microbiol](javascript:AL_get(this, 'jour', 'J Clin Microbiol.');) 2006;44:3366-7.
35. Cherian T, Bobo L, Steinhoff MC, Karron RA, Yolken RH. Use of PCR- enzyme immunoassay for identification of influenza A virus matrix RNA in clinical samples negative for cultivable virus. J Clin Microbiol 1994; 32: 623–628.
36. American Academy of Pediatrics Subcommittee on Management of Acute Otitis Media. Diagnosis and management of acute otitis media. Pediatrics 2004; 113:1451-65.
37. Brown PD and Lerner SA. Community-acquired pneumonia. Lancet 1998; 352: p. 1295-302.
38. Ontario Case Costing Project (OCCP). Toronto, Ontario: Ontario Ministry of Health and Long-term Care, 2005. (Accessed November 25, 2005 at [http://www.occp.com](http://www.occp.com/)).
39. Campbell MJ, Donner A, Klar A. Developments in cluster randomized trials and Statistics in Medicine. Stat Med 2007; 26:2-19.
40. Hoberman A, Greenberg DP, Paradise JL, et al. Effectiveness of inactivated influenza vaccine in preventing acute otitis media in young children: a randomized controlled trial.
    JAMA 2003; 290:1608-16.
41. Kerry SM and Bland JM. Unequal cluster sizes for trials in English and Welsh general practice: implications for sample size calculations. Statistics in Medicine 2001; 20: 377-390.
42. Loeb M, Craven S, McGeer A, Simor AE, Bradley S, Low DE, Moss L, Armstrong-Evans M, Walter S. **Risk factors for resistance to antimicrobial agents among nursing home residents**. Am J Epidemiol 2003; 157:40-47.
43. Loeb M, Brazil K, Lohfeld L, McGeer A, Simor AE, Stevenson K, Zoutman D, Liu X, Smith S, Walter S. Effect of a multifaceted intervention on number of antimicrobial prescriptions for suspected urinary tract infections in residents of nursing homes: cluster randomized controlled trial. BMJ 2005; 331:669.
44. Loeb M, Chan Carusone S, Goeree R, Walter S, Brazil K, Krueger P, Simor A, Moss L, Marrie T. Effect of a clinical pathway for reducing hospitalization among residents of nursing homes with pneumonia: a randomized controlled trial. JAMA 2006; 295:2503-10.
45. Mills E, Gold R, Thipphawong J, Barreto L, Guasparini R, Meekison W, Cunning L, Russell M, Harrison D, Boyd M, Xie F. Safety and immunogenicity of a combined five-component pertussis-diphtheria-tetanus-inactivated poliomyelitis-*Haemophilus B* conjugate vaccine administered to infants at two, four and six months of age. Vaccine 1998; 16:576-585.
46. Halperin SA, Smith B, Russell M, Scheifele D, Mills E, Hasselback P, Pim C, Meekison W, Parker R, Lavigne P, Barreto L. An adult formulation of a five-component acellular pertussis vaccine combined with diphtheria and tetanus toxoids and inactivated poliovirus vaccine is safe and immunogenic in adolescents and adults. Vaccine 2000; 18:1312-9.
47. Skowronski DM, De Serres G, Scheifele D, Russell ML, Warrington R, Davies HD, Dionne M, Duval B, Kellner J, MacDonald J. Randomized, double-blind, placebo-controlled trial to assess the rate of recurrence of oculorespiratory syndrome following influenza vaccination among persons previously affected. Clin Infect Dis 2003; 37:1059-66.
48. [Nicholson KG, Aoki FY, Osterhaus AD,](http://www.ncbi.nlm.nih.gov/entrez/query.fcgi?db=pubmed&cmd=Retrieve&dopt=AbstractPlus&list_uids=10866439&query_hl=3&itool=pubmed_docsum) Efficacy and safety of oseltamivir in treatment of acute influenza: a randomised controlled trial. Neuraminidase Inhibitor Flu Treatment Investigator Group. Lancet 2000; 355:1845-50.
49. [Lee BE, Robinson JL, Khurana V, Pang XL, Preiksaitis JK, Fox JD.](http://www.ncbi.nlm.nih.gov/entrez/query.fcgi?db=pubmed&cmd=Retrieve&dopt=AbstractPlus&list_uids=16555283&query_hl=8&itool=pubmed_docsum) Enhanced identification of viral and atypical bacterial pathogens in lower respiratory tract samples with nucleic acid amplification tests. J Med Virol 2006; 78:702-10.
50. Zakhartchouk AN, Liu Q, Petric M, Babiuk LA. Augmentation of immune responses to SARS coronavirus by a combination of DNA and whole killed virus vaccines. Vaccine 2005; 23(35): 4385-91.
51. Dushoff J, Plotkin JB, Levin S, Earn DJD. Dynamical resonance can account for seasonality of influenza epidemics. Proceedings of the National Academy of Sciences of the USA 2004; 101: 16915-16916.
52. Earn DJD, Dushoff J, Levin SA. Ecology and evolution of the flu. Trends in Ecology and Evolution 2002; 17: 334-340.
53. Earn DJD, Rohani P, Bolker BM, Grenfell BT. A simple model for complex dynamical transitions in epidemics. Science 2000; 287: 667-670.
54. Walter SD, Awasthi S, Jeyaseelan L. Pre-trial evaluation of the potential for unblinding in drug trials: a prototype example. Contemporary Clinical Trials 2005; 26: 459-68.
55. Chan SF, Macaskill P, Irwig L, Walter SD. Adjustment for baseline measurement error in randomized controlled trials induces bias. Controlled Clinical Trials 2004; 25: 408-16.

**Figure 1.** Excess death from all causes and excess death due to pneumonia and influenza1950-1998, Japan and US (16).


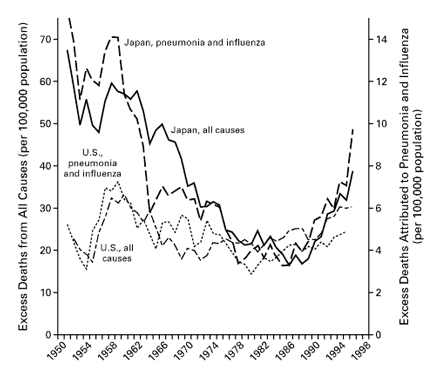


**Figure 2.** Map of Hutterite colonies in David Thompson region

**Figure 3:** Study flow diagram

138 Hutterite colonies approached

36 colonies ineligible

- 12: high risk < 10

- 3: children immunized because of a high risk child (cystic fibrosis)

- 7: geographic distance > 150 km

- 14: do not vaccinate children

30 colonies declined to participate

- 25: not interested

- 5: colony is splitting

72 eligible Hutterite colonies

*****Alberta health regions (CA=Calgary, DT=David Thompson, CH=Chinook, EC=East Central), Saskatchewan health regions (CY/FH=Cypress/Five Hills, HL=Heartland), and Manitoba health regions (CE=Central, AS=Assiniboine).

Alberta

Manitoba

Saskatchewan

CA

n=8

DT

n=4

CY/FH

n=8

HL

n=6

CE

n=6

AS

n=6

CH

n=14

EC

n=8

I=4

C=4

I=2

C=2

I=7

C=7

I=4

C=4

I=4

C=4

I=3

C=3

I=3

C=3

I=3

C=3

Provinces

Health* regions

(n = colonies)

No. Intervention colonies

No. Control colonies

60 colonies to be randomized

**Figure 4.** Distribution of eligible Hutterite colonies in Alberta, Saskatchewan, and Manitoba willing to participate in the study.

**
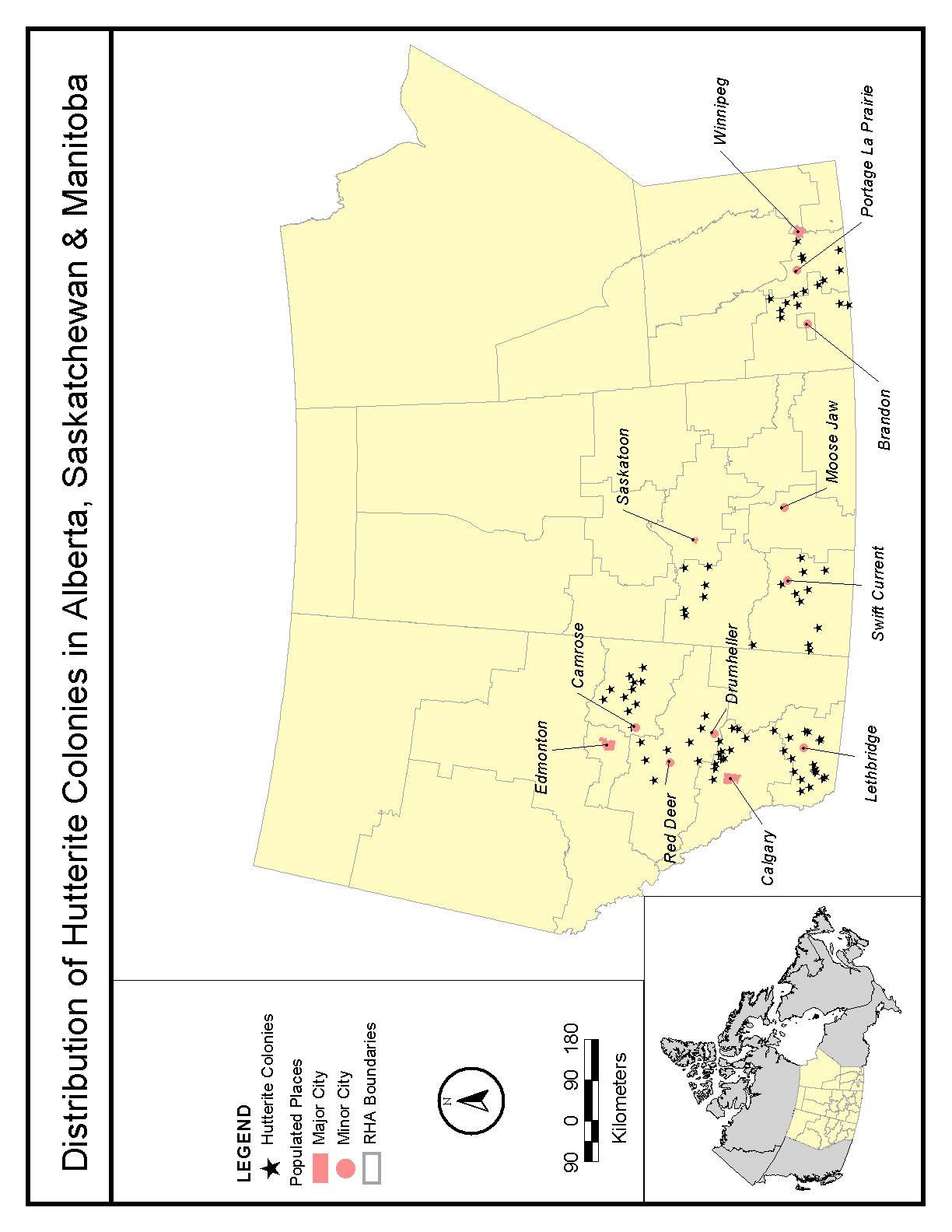
**

**Figure 5:** Sequence of laboratory testing

NP swab (>2 years of age)

or throat swabs collected from Participants

PROVINCIAL LABS:

PCR Influenza A/ B

Positive

Negative

Viral culture

NATIONAL MICROBIOLOGY LAB:

Viral serotyping

**Figure 6:** Study administration and structure

Data Safety & Monitoring Board

**Coordinating Centre**

(McMaster University)

Manitoba Provincial Centre

(University of Manitoba)

Cypress Health Region

(Saskatchewan)

Alberta Provincial Centre

(University of Calgary)

Five Hills Health Region

(Saskatchewan)

Alberta Provincial Lab

(Calgary)

Saskatchewan Provincial Lab

(Regina)

Cadham Provincial Lab

(Winnipeg)

National Microbiology Laboratory

(Winnipeg, Manitoba)

**Table 1.** Characteristics of eligible Hutterite colonies.

| **CHARACTERISTIC** | **Study provinces** | | | **Total** |
| --- | --- | --- | --- | --- |
|  | **Alb.** | **Sask.** | **Man.** |  |
| Number of colonies | 38 | 17 | 17 | 72 |
| Number of high risk participants | 581 | 306 | 436 | **1323** |
| Number ≥ 65 yrs | 198 | 91 | 86 | 375 |
| Number ≥ 6 mo ≤23 mo | 165 | 96 | 121 | 382 |
| Number with cardiopulmonary conditions | 30 | 9 | 55 | 94 |
| Number with metabolic conditions | 89 | 51 | 46 | 186 |
| Number with neurological conditions | 0 | 3 | 5 | 8 |
| Number with other high risk conditions | 29 | 17 | 90 | 136 |
| Number of healthy children between 36 mo. to 15 yrs | 920 | 430 | 408 | **1758** |
| Number of other colony members | 2005 | 793 | 728 | **3526** |
| Total number of eligible participants | 3506 | 1529 | 1572 | **6607** |

**Table 2.A. Schedule of active and placebo vaccination for participants < 9 years at year 1***

| Study Year | Influenza Arm | | Hepatitis Arm | | |
| --- | --- | --- | --- | --- | --- |
|  | Week 1 | Week 4 | | Week 1 | Week 4 |
| Year 1 | V | V | | A | S |
| Year 2 | V |  | | A |  |
| Year 3* | V |  | | S |  |

**Table 2.B.** Schedule of active and placebo vaccination for participants ≥ 9 years at year 1*

| Study Year | Influenza Arm | | Hepatitis Arm | | |
| --- | --- | --- | --- | --- | --- |
|  | Week 1 | Week 4 | | Week 1 | Week 4 |
| Year 1 | V |  | | A |  |
| Year 2 | V |  | | A |  |
| Year 3* | V |  | | S |  |

*V denotes VAXIGRIP® vaccine, A denoted AVAXIM®, S denotes physiologic saline.

* Individuals immunized with AVAXIM for the first time in Year 3 will receive a booster 12 months later after completion of the trial. Individuals who received the first dose of hepatitis the previous year will receive second dose in year 3.

**Table 3.** Power calculations for ICC’s ranging from 0.01 to 0.05, assuming either a 10% or 8% attack rate and relative risk reduction of 50%, two-sided alpha of 0.05, 60 clusters, and cluster size of 50.

| *Control rate* | *Intervention*  *rate* | *Cluster*  *size* | *Colonies*  *Total* | *Participants per group* | *ICC* | *Power* |
| --- | --- | --- | --- | --- | --- | --- |
| 0.10 | 0.05 | 50 | 60 | 1500 | 0.01 | 99% |
| 0.10 | 0.05 | 50 | 60 | 1500 | 0.02 | 96% |
| 0.10 | 0.05 | 50 | 60 | 1500 | 0.03 | 91% |
| 0.10 | 0.05 | 50 | 60 | 1500 | 0.04 | 85% |
| 0.10 | 0.05 | 50 | 60 | 1500 | 0.05 | 80% |
| 0.08 | 0.04 | 50 | 60 | 1500 | 0.01 | 90% |
| 0.08 | 0.04 | 50 | 60 | 1500 | 0.02 | 90% |
| 0.08 | 0.04 | 50 | 60 | 1500 | 0.03 | 83% |
| 0.08 | 0.04 | 50 | 60 | 1500 | 0.04 | 76% |

**Table 4: Study calendar**

| **Activities** | **08/2008-07/2009** | | | | | | **08/2009-07/2010** | | | | | | **08/2010-07/2011** | | | | | |
| --- | --- | --- | --- | --- | --- | --- | --- | --- | --- | --- | --- | --- | --- | --- | --- | --- | --- | --- |
|  | 08 09 | 11 | 01 | 03 | 05 | 07 | 08 09 | 11 | 01 | 03 | 05 | 07 | 08 09 | 11 | 01 | 03 | 05 | 07 |
| Training of nurses | 🗸 |  |  |  |  |  |  |  |  |  |  |  |  |  |  |  |  |  |
| Allocation of intervention | 🗸 |  |  |  |  |  |  |  |  |  |  |  |  |  |  |  |  |  |
| Enrolling healthy children | 🗸 |  |  |  |  |  | 🗸 |  |  |  |  |  | 🗸 |  |  |  |  |  |
| Enrolling other participants | 🗸 |  |  |  |  |  | 🗸 |  |  |  |  |  | 🗸 |  |  |  |  |  |
| Pre season serology | 🗸 |  |  |  |  |  | 🗸 |  |  |  |  |  | 🗸 |  |  |  |  |  |
| Vaccination of children |  | 🗸 |  |  |  |  |  | 🗸 |  |  |  |  |  | 🗸 |  |  |  |  |
| Post vaccine serology |  | 🗸 |  |  |  |  |  | 🗸^ß^ |  |  |  |  |  | 🗸 ^*^ |  |  |  |  |
| Follow-up** † |  | 🗸 | 🗸🗸 | 🗸🗸 |  |  |  | 🗸 | 🗸🗸 | 🗸🗸 |  |  |  | 🗸 | 🗸🗸 | 🗸🗸 |  |  |
| Mid season  serology |  |  | 🗸 |  |  |  |  |  | 🗸 |  |  |  |  |  | 🗸 |  |  |  |
| Post season serology* |  |  |  |  | 🗸 |  |  |  |  |  | 🗸 |  |  |  |  |  | 🗸 |  |
| Data Collection |  | 🗸 | 🗸🗸 | 🗸🗸 | 🗸🗸 |  |  | 🗸 | 🗸🗸 | 🗸🗸 | 🗸🗸 |  |  | 🗸 | 🗸🗸 | 🗸🗸 | 🗸🗸 |  |
| Yearly reports to DSMB |  |  | 🗸 |  |  |  |  |  | 🗸 |  |  |  |  |  | 🗸 |  |  |  |
| End of Season report |  |  |  |  |  | 🗸 |  |  |  |  |  | 🗸 |  |  |  |  |  | 🗸 |
| Data analysis |  |  |  |  |  |  |  |  |  |  |  |  |  |  |  |  |  | 🗸🗸 |
| Manuscript preparation |  |  |  |  |  |  |  |  |  |  |  |  |  |  |  |  |  | 🗸🗸 |

*Second and third year blood will be collected from the vaccine group to confirm children receiving hepatitis A have developed protective titres.

**Follow-up and post-season serology dates will be determined by the influenza activity within the health region.

†- Acute and convalescent blood samples will be collected from health vaccinated children as required.

**Table 5:** Schedule of events and study forms

| **Data Collected, or Event** | **Enrolment**  **& first blood draw** | **Vaccination** | **Post-Vaccination**  Vaccination + 3 d | **Second Vaccination & blood draw**  Vaccination + 1 mo | **Follow-up Visits**  Start to end of influenza season (2xs/wk) | **Mid-Season Blood Draw** | **Final blood draw**  End of Influenza season |
| --- | --- | --- | --- | --- | --- | --- | --- |
| Informed Consent | X |  |  |  |  |  |  |
| Screening and Enrolment Log  (Form 00) | X |  |  |  |  |  |  |
| Influenza Visit Log  (Form 01) | X | X | X | X | X | X | X |
| Eligibility Form (Form 10) | X |  |  |  |  |  |  |
| Blood draw for HI | X  HR |  |  | X  HR* Yr 2 & 3 VG |  | X  HR | X  HR |
| Influenza Serology Log  (Form 02) | X  HR |  |  | X  HR* VG |  | X  HR | X  HR |
| Vaccination Questionnaire  (Form 20) |  | X  VG |  | X  VG (<9**) |  |  |  |
| Vaccination |  | X  VG |  | X  VG (<9**) |  |  |  |
| Vaccination Log  (Form 03) |  | X  VG |  | X  VG (<9**) |  |  |  |
| Post-Vaccination Form  (Form 21) |  |  | X  VG |  |  |  |  |
| Serious Adverse Event Reporting Form (Form 22) |  | As needed  VG | As needed  VG | As needed  VG (<9**) |  |  |  |
| Adverse Event Observation Form  (Form 23) |  | As needed  VG | As needed  VG | As needed  VG |  |  |  |
| Follow-up Form  (Form 30) |  |  |  |  | X  ALL GROUPS |  |  |
| Respiratory Infection Form  (Form 31) |  |  |  |  | As needed  ALL GROUPS |  |  |
| Nasopharyngeal Swab/Throat Swab |  |  |  |  | As needed  ALL GROUPS |  |  |
| Nasopharyngeal Swab Log  (Form 04) |  |  |  |  | As needed  ALL GROUPS |  |  |
| Acute and convalescent serology |  |  |  |  | As needed  VG |  |  |
| Primary Care Data Collection Form (Form 40) |  |  |  |  | As needed  ALL GROUPS |  |  |
| Hospitalization Data Collection Form (Form 41) |  |  |  |  | As needed  ALL GROUPS |  |  |
| Discontinuation Report  (Form 50) |  | As needed | As needed | As needed | As needed | As needed | As needed |

ALL GROUPS= VG (vaccination group) and OH (other group), including HR (high risk group- other sub group)

* HR individuals who have received a flu shot, ** Individuals in the vaccination group who are < 9 years old and have not had a previous flu shot
